# Multiple Plant Surface Signals are Sensed by Different Mechanisms in the Rice Blast Fungus for Appressorium Formation

**DOI:** 10.1371/journal.ppat.1001261

**Published:** 2011-01-20

**Authors:** Wende Liu, Xiaoying Zhou, Guotian Li, Lei Li, Lingan Kong, Chenfang Wang, Haifeng Zhang, Jin-Rong Xu

**Affiliations:** 1 Purdue-NWAFU Joint Research Center, Northwest A&F University, Yangling, Shaanxi, China; 2 Department of Botany and Plant Pathology, Purdue University, West Lafayette, Indiana, United States of America; University of Melbourne, Australia

## Abstract

Surface recognition and penetration are among the most critical plant infection processes in foliar pathogens. In *Magnaporthe oryzae*, the Pmk1 MAP kinase regulates appressorium formation and penetration. Its orthologs also are known to be required for various plant infection processes in other phytopathogenic fungi. Although a number of upstream components of this important pathway have been characterized, the upstream sensors for surface signals have not been well characterized. Pmk1 is orthologous to Kss1 in yeast that functions downstream from Msb2 and Sho1 for filamentous growth. Because of the conserved nature of the Pmk1 and Kss1 pathways and reduced expression of *MoMSB2* in the *pmk1* mutant, in this study we functionally characterized the *MoMSB2* and *MoSHO1* genes. Whereas the *Momsb2* mutant was significantly reduced in appressorium formation and virulence, the *Mosho1* mutant was only slightly reduced. The *Mosho1 Momsb2* double mutant rarely formed appressoria on artificial hydrophobic surfaces, had a reduced Pmk1 phosphorylation level, and was nonresponsive to cutin monomers. However, it still formed appressoria and caused rare, restricted lesions on rice leaves. On artificial hydrophilic surfaces, leaf surface waxes and primary alcohols-but not paraffin waxes and alkanes- stimulated appressorium formation in the *Mosho1 Momsb2* mutant, but more efficiently in the *Momsb2* mutant. Furthermore, expression of a dominant active *MST7* allele partially suppressed the defects of the *Momsb2* mutant. These results indicate that, besides surface hydrophobicity and cutin monomers, primary alcohols, a major component of epicuticular leaf waxes in grasses, are recognized *by M. oryzae* as signals for appressorium formation. Our data also suggest that MoMsb2 and MoSho1 may have overlapping functions in recognizing various surface signals for Pmk1 activation and appressorium formation. While MoMsb2 is critical for sensing surface hydrophobicity and cutin monomers, MoSho1 may play a more important role in recognizing rice leaf waxes.

## Author Summary

The rice blast fungus is a major pathogen of rice and a model for studying fungal-plant interactions. Like many other fungal pathogens, it can recognize physical and chemical signals present on the rice leaf surface and form a highly specialized infection structure known as appressorium. A well conserved signal transduction pathway involving the protein kinase gene *PMK1* is known to regulate appressorium formation and plant penetration in this pathogen. However, it is not clear about the sensor genes that are involved in recognizing various plant surface signals. In this study we functionally characterize two putative sensor genes called *MoMSB2* and *MoSHO1*. Genetic and biochemical analyses indicated that these two genes have overlapping functions in recognizing different physical and chemical signals present on the rice leaf surface for the activation of the Pmk1 pathway and appressorium formation. We found that primary alcohols, a major component of leaf waxes in grasses, can be recognized by the rice blast fungus as chemical cues. While *MoMSB2* is critical for sensing hydrophobicity and precursors of cutin molecules of rice leaves, *MoSHO1* appears to be more important than *MoMSB2* for recognizing wax components.

## Introduction

The heterothallic ascomycete *Magnaporthe oryzae* is an important pathogen of rice throughout the world. In the past two decades, the rice-*M. oryzae* pathosystem has been developed as a model to study fungal-plant interactions [1], [2], [3]. *M. oryzae* initiates infection of rice leaves by the germination of conidia and differentiation of appressoria at the tip of germ tubes. The fungus then uses turgor pressure that develops within appressoria to penetrate the plant cuticle and cell wall. After penetration, the narrow penetration peg differentiates into invasive hyphae, which are enveloped by the host cytoplasmic membrane during the biotrophic phase [4]. As a hemibiotrophic pathogen, *M. oryzae* does not kill plant cells initially. At late infection stages, plant cells are killed due to extensive growth of infectious hyphae and blast lesions are normally visible within 7 days post-infection.

The surface of rice leaves is comprised of epicuticular waxes. Germ tubes of *M. oryzae* recognize the hydrophobicity of rice leaves. The fungus also forms appressoria on artificial hydrophobic surfaces. On hydrophilic surfaces, conidia produce long germ tubes without tip differentiation. Exogenous cAMP induces appressorium formation on hydrophilic surfaces. Molecular studies have confirmed the role of cAMP signaling in surface recognition and initiation of appressorium formation [5], [6]. Besides surface hydrophobicity, other factors including surface hardness, cutin monomers, and leaf waxes also affect appressorium formation in *M. oryzae* [7], [8], [9], [10], [11]. Various physical and chemical signals also have been shown to affect appressorium formation in other plant pathogenic fungi, including *Ustilago maydis* and *Colletotrichum* species.

While cAMP signaling controls surface recognition and tip deformation, the Pmk1 MAP kinase pathway regulates late stages of appressorium formation, penetration, and infectious growth in *M. oryzae* [12]. Pmk1 is orthologous to Kss1, which is a key MAP kinase involved in the filamentous growth pathway in *Saccharomyces cerevisiae* [13]. A number of genes functioning upstream from Pmk1, including the MEK (Mst7) and MEK kinase (Mst11), an adaptor protein Mst50, and Ras2 have been identified [14], [15]. One of the downstream transcription factors regulated by Pmk1 is Mst12, which is required for appressorial penetration and invasive growth [16]. The Pmk1 pathway is conserved in phytopathogenic fungi for regulating various infection processes [15]. Although key components of the cAMP signaling and Pmk1 pathways have been identified, the mechanisms for recognizing physical and chemical signals of plant surfaces have not been well studied in *M. oryzae* and other fungal pathogens. One putative receptor gene known to be involved in surface sensing is *PTH11* [12], [17], [18]. The *pth11* mutant is reduced in virulence and appressorium formation on hydrophobic surfaces, but it forms abundant appressoria in the presence of exogenous cAMP [19]. The *M. oryzae* genome contains about 60 putative GPCR genes, including several *PTH11*-like genes with the CEFM domain.

The filamentation MAPK pathway is well studied in yeast [20]. The downstream target of Kss1, Ste12, forms a heterodimer with Tec1 to regulate the expression of genes related to filamentous growth. Various genes, including *RAS1*, *CDC42*, *SHO1*, and *MSB2*, function upstream from the Kss1-dependent filamentation pathway [21], [22]. *MSB2* encodes a surface mucin protein that interacts with Cdc42 for filamentous growth. Msb2 also interacts with Hkr1 and functions upstream from Sho1 for responses to hyperosmotic stresses [22]. *SHO1* encodes a membrane sensor protein that is involved in the activation of the Ste11-Ste7-Kss1 pathway and the Hog1 osmoregulation pathway in yeast [21], [23]. During the preparation of this manuscript, *msb2* and *sho1* were shown to be essential for regulating appressorium formation and tumor development in the corn smut fungus *Ustilago maydis* [24]. The *msb2 sho1* mutant failed to form appressoria on artificial and plant surfaces and was non-pathogenic.

Because of the conserved nature of the Pmk1 and Kss1 MAPK cascades and the importance of *MSB2* and *SHO1* in yeast filamentous growth, we examined the expression levels of their orthologs in *M. oryzae* (named *MoMSB2* and *MoSHO1* in this study). Both of them were reduced over 4-fold in the *pmk1* mutant. Deletion of *MoSHO1* had only minor effects on appressorium formation, but the *Momsb2* deletion mutants rarely formed appressoria on artificial hydrophobic surfaces and failed to respond to cutin monomers. However, they were still pathogenic and recognized leaf surface waxes for appressorium formation. Further analyses indicated that primary alcohols in rice leaf epicuticular waxes induce appressorium formation. Expression of a dominant active allele of *MST7* [14] also stimulated appressorium development in the *Momsb2* mutant, which had a reduced level of Pmk1 phosphorylation. Overall, these results show that primary alcohols, a major component of epicuticular leaf waxes in grasses, are recognized by *M. oryzae* as signals for appressorium formation. Other fungal pathogens may also recognize primary alcohols or other wax components. Our data also indicate that MoMsb2 and MoSho1 may function as upstream sensors for the activation of the Pmk1 pathway and appressorium formation. While MoMsb2 is critical for sensing surface hydrophobicity and cutin monomers, MoSho1 may plays a more important role than *MoMSB2* in recognizing rice leaf waxes.

## Results

### The *MSB2* and *SHO1* orthologs in *Magnaporthe oryzae*

**Figure 1:**
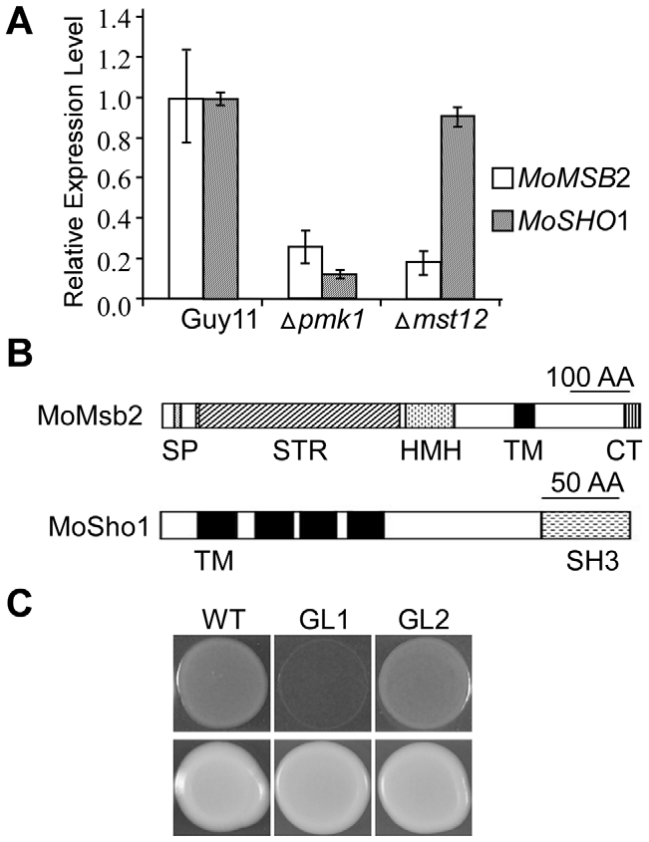
The *MSB2* and *SHO1* orthologs in *M. oryzae*. **A**. qRT-PCR assay of *MoMSB2* and *MoSHO1* expression in the wild-type, *pmk1*, and *mst12* mutant strains. The relative expression level of *MoMSB2* and *MoSHO1* in the mutants was compared to that of the wild-type strain (arbitrarily set to 1). Mean and standard error were calculated with data from three biological replicates. **B**. Schematic drawing of domains identified in MoMsb2 and MoSho1. SP, signal peptide; STR, serine/threonine rich region; HMH, Hkr1-Msb2 homology domain; TM, transmembrane domain; CT, cytoplasmic tail; SH3, and Src homology 3 domain. **C**. Colonies of XK1-25 (WT) and *sho1* mutant transformed with pYES2 (GL1) or pMoSHO1 (GL2). Photos were taken after incubation for two days on YPGal plates with or without 1.5 M sorbitol.

Like other filamentous fungi, *M. oryzae* has one distinct ortholog each for the yeast *MSB2* and *SHO1* genes (Fig. S1), which were named *MoMSB2* and *MoSHO1*, respectively. When assayed by qRT-PCR with three independent biological replicates, both *MoMSB2* and *MoSHO1* transcripts had reduced expression levels (over 4-fold) in the *pmk1* mutant but only *MoMSB2* expression was significantly reduced in the *mst12* mutant (Fig. 1A). The MoMsb2 protein has a signal peptide and one C-terminal transmembrane (TM) domain (Fig. 1B). Therefore, only the C-terminal portion behind the TM domain is inside the cytoplasm membrane. The bulk of the mature MoMsb2 protein is predicted to be extracellular and contains numerous putative glycosylation sites. In yeast, the promoter of *MSB2* has two pheromone response elements (PRE) recognized by Ste12 and one TEA/ATTS consensus sequence (TCS) recognized by Tec1 [25]. The promoter region of *MoMSB2* has two PRE-like sequences and two putative TCS elements (Fig. S2).

Like *MoMSB2*, *MoSHO1* is well conserved in filamentous fungi. It encodes a protein with four TM and one SH3 domains (Fig. 1B). Although MoSho1 and Sho1 share only 34% identity in amino acid sequences, expression of *MoSHO1* in yeast functionally complemented the defects of the *sho1* mutant in growth on medium with 1.5 M sorbitol (Fig. 1C). When *MoMSB2* was expressed in yeast, similar to the *msb2* gene from *U. maydis* [24], it failed to complement filamentation defects of the *msb2* mutant.

### *MoMSB2* and *MoSHO1* are involved in recognizing artificial hydrophobic surfaces

To determine their functions in *M. oryzae*, we used the gene replacement approach to delete the *MoMSB2* and *MoSHO1* genes. One *Mosho1* and two *Momsb2* mutants (Table 1) were identified and confirmed by Southern blot analysis (Fig. S3). The two *Momsb2* mutants had the same phenotype although only data for mutant M6 were presented here. The *Mosho1* mutant had no obvious defects in vegetative growth, but the growth rate of the *Momsb2* mutant was slightly reduced (Table 2) in comparison with that of Ku80 [26], which was used to generate the mutants.

**Table 1:** Wild-type and mutant strains of *Magnaporthe oryzae* used in this study.

Strain	Genotype description	Reference
Guy11	Wild-type (*MAT1-2, avr-Pita*)	Chao and Ellingboe, 1991
70-15	Wild-type (*MAT1-1, AVR-Pita*)	Chao and Ellingboe, 1991
Ku80	*MgKu80* deletion mutant of Guy11	Villalba et al., 2008
nn78	*pmk1* mutant	Xu and Hamer, 1996
mk23	*mst12* mutant	Park et al., 2004
M2	*Momsb2* deletion mutant	This study
M6	*Momsb2* deletion mutant	This study
S72	*Mosho1* deletion mutant	This study
MS88	*Mosho1 MoMsb2* double mutant	This study
MS93	*Mosho1 MoMsb2* double mutant	This study
CM6	Complemented strain of M6 (*Momsb2*/*MoMSB2*-eGFP)	This study
Cs12	Transformant of 70-15 expressing *MoSHO1*-eGFP	This study
CS15	Complemented strain of S72 (*Mosho1*/*MoSHO1*)	This study
CMS74	*Mosho1 MoMsb2*/*MoSHO1 MoMSB2* complemented strain	This study
Ect7	Ectopic transformant for *MoSHO1* deletion	This study
Ect12	Ectopic transformant for *MoSHO1* deletion	This study
Ect16	Ectopic transformant for *MoMSB2* deletion	This study
Ect20	Ectopic transformant for *Mosho1 MoMsb2* deletion	This study
DSSM	Transformant of M6 expressing *MoMSB2* ^ΔSP^-eGFP	This study
WDA2	Transformant of 70-15 expressing the *MST7* ^DA^ allele	This study
WDA12	Transformant of M6 expressing the *MST7* ^DA^ allele	This study

**Table 2:** Phenotype characterization of the mutants generated in this study.

Strain	Growth rate (mm/day)^a^	Conidiation (×10^6^ spores/plate)	Appressorium formation (%)^b^	Lesion formation^c^ (lesions/5cm leaf tip)
Ku80	5.6±0.1 A*	23.3±5.4 A	99.4±0.5 A	24.8±14.7 A
M6	5.0±0.1 B	22.9±5.5 A	1.5±0.5 D	2.1±1.1 C
S72	5.2±0.1 B	4.1±0.8 C	72.8±5.2 B	9.2±5.3 B
MS88	5.0±0.1 B	2.8±0.5 C	0.9±0.4 D	0.9±0.8 C
Ect20	5.7±0.3 A	4.0±0.4 C	98.5±0.4 A	N/A^d^
CM6	5.5±0.2 A	21.5±2.0 A	96.0±2.6 A	N/A
CS15	5.6±0.2 A	11.3±2.5 B	96.7±2.1 A	N/A
CMS74	5.6±0.2 A	11.0±3.0 B	95.3±1.5 A	N/A

a Growth rate (extension in colony diameter) and conidiation were assayed with OA cultures.

b Percentage of germ tubes formed appressoria on the hydrophobic side of Gelbond membranes by 24 h.

c Lesion formation was examined on infected rice leaves 7 days after inoculation. Means and SD values were calculated from at least three independent experiments.

d Not assayed.

*Data from three replicates were analyzed with the protected Fisher's Least Significant Difference (LSD) test. The same letter indicated that there was no significant difference. Different letters were used to mark statistically significant difference (P = 0.05).

**Figure 2:**
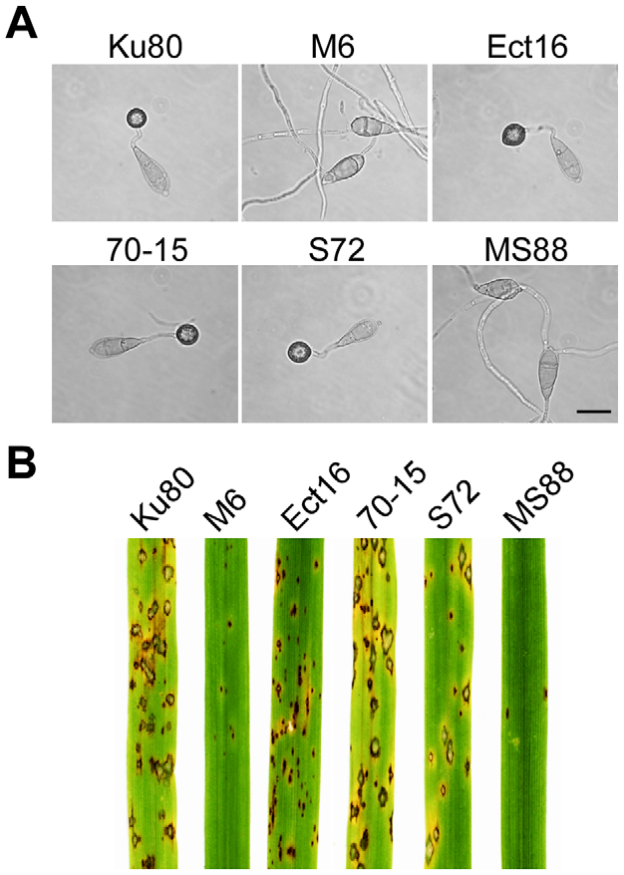
Appressorium formation and plant infection assays. **A**. Conidia from wild-type strains 70-15 and Ku80, *Momsb2* mutant M6, *Mosho1* mutant S72, *Mosho1 Momsb1* mutant MS88, and an ectopic transformant Ect16 were incubated on hydrophobic surfaces for 24 h. Bar = 20 µm. **B**. Rice leaves sprayed with conidia from the same set of strains. Typical leaves were photographed 7 dpi.

On hydrophobic surfaces, both the *Momsb2* and *Mosho1* mutants had no defects in conidium germination. However, the *Momsb2* mutant was significantly reduced in appressorium formation. Less than 2% of its germ tubes formed melanized appressoria by 24 h (Fig. 2A). Under the same conditions, over 90% and 70% of the germ tubes formed appressoria in Ku80 and the *Mosho1* mutant, respectively (Table 2), indicating that the *Mosho1* mutant was only slightly reduced in appressorium formation.

Because of the functions of Sho1 and Msb2 in yeast filamentous growth [22] and reduced appressorium formation in both *Mosho1* and *Momsb2* mutants, we deleted the *MoMSB2* gene in the *Mosho1* mutant. Transformants MS88 and MS93 (Table 1) were two *Mosho1 Momsb2* mutants confirmed by Southern analysis (Fig. S3). On the hydrophobic surfaces, the double mutant produced long, curved germ tubes (Fig. 2A) that rarely (<1%) differentiated into appressoria (Table 2). These results indicate that *MoMSB2* plays a critical role but *MoSHO1* also plays a minor role in the recognition of surface hydrophobicity.

### The *Momsb2* and *Momsb2 Mosho1* mutants are reduced in virulence

In infection assays with rice seedlings, the *Mosho1* mutant was only slightly reduced in virulence (Fig. 2B, Table 2). In contrast, the *Momsb2* and *Mosho1 Momsb2* mutants were significantly reduced in virulence. Only rare lesions were observed on leaves inoculated with the double mutant (Table 2). The *Momsb2* mutant caused a few more lesions than the double mutant MS88 but still much less than Ku80 and the *Mosho1* mutant (Table 2). Lesions caused by the *Momsb2* and *Mosho1 Momsb2* mutants on rice leaves tended to be smaller than those caused by Ku80 (Fig. 2B) and have limited necrotic borders (Fig. S4).

Similar results were obtained in barley infection assays. Ku80 and the *Mosho1* mutant caused numerous lesions on inoculated leaves. Under the same conditions, only a few lesions were formed on barley leaves infected with the *Momsb2* and *Momsb2 Mosho1* mutants, indicating a significant reduction in virulence. Results from these infection assays indicate that *MoMSB2* plays a more critical role than *MoSHO1* in pathogenesis. However, *MoSHO1* also is required for full virulence because the *Mosho1* mutant had reduced virulence (Table 2) and the *Momsb2* mutant appeared to be more virulent than the *Momsb2 Mosho1* double mutant.

For complementation assays, we re-introduced the wild-type *MoSHO1* and *MoMSB2* alleles to the mutants. Transformants CM6 (*Momsb2*/*MoMSB2*), CS15 (*Mosho1*/*MoSHO1*), and CMS74 (*Mosho1 Momsb2*/*MoSHO1 MoMSB2*) were normal in virulence (Fig. S5), vegetative growth, and appressorium formation (Table 2), indicating that reintroduction of the wild-type *MoSHO1* and *MoMSB2* genes complemented the defects of corresponding mutants.

### Deletion of *MoMSB2* does not affect appressorium formation on plant leaves

**Figure 3:**
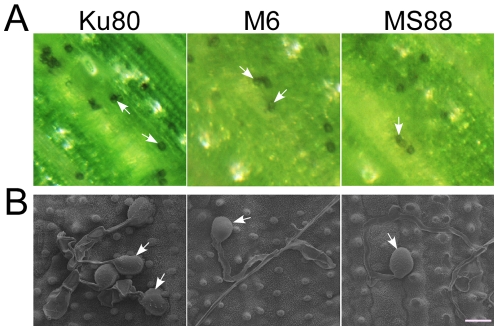
Appressorium formation assays with intact rice leaves. **A**. Rice leaves inoculated with conidia from strains Ku80, M6 (*Momsb2*), and MS88 (*Mosho1 Momsb2*). Melanized appressoria were observed 24 hpi. **B**. Appressoria formed by the same set of strains on rice leaf surface examined under SEM. The mutants produced appressoria at the tip of long germ tubes. Bar = 10 µm. Arrows marked appressoria.

Because the double mutant rarely formed appressoria on artificial surfaces but still caused blast lesions, we assayed its ability to form appressoria on rice leaves. At 24 h, the *Momsb2* and *Momsb2 Mosho1* mutants produced abundant melanized appressoria (Fig. 3A). When examined by scanning electron microscopy (SEM), 68.7±7.3% and 57.7±8.4% of the *Momsb2* and *Mosho1 Momsb2* germ tubes, respectively, formed appressoria (Fig. 3B). Similar results were obtained in appressorium formation assays with barley leaves (Fig. S6), indicating that the *Momsb2* and *Mosho1 Momsb2* mutants still respond to chemical cues present on rice and barley leaf surfaces for appressorium formation.

### Cutin monomers fail to induce appressorium formation in the *Momsb2* and *Momsb2 Mosho1* mutants

**Figure 4:**
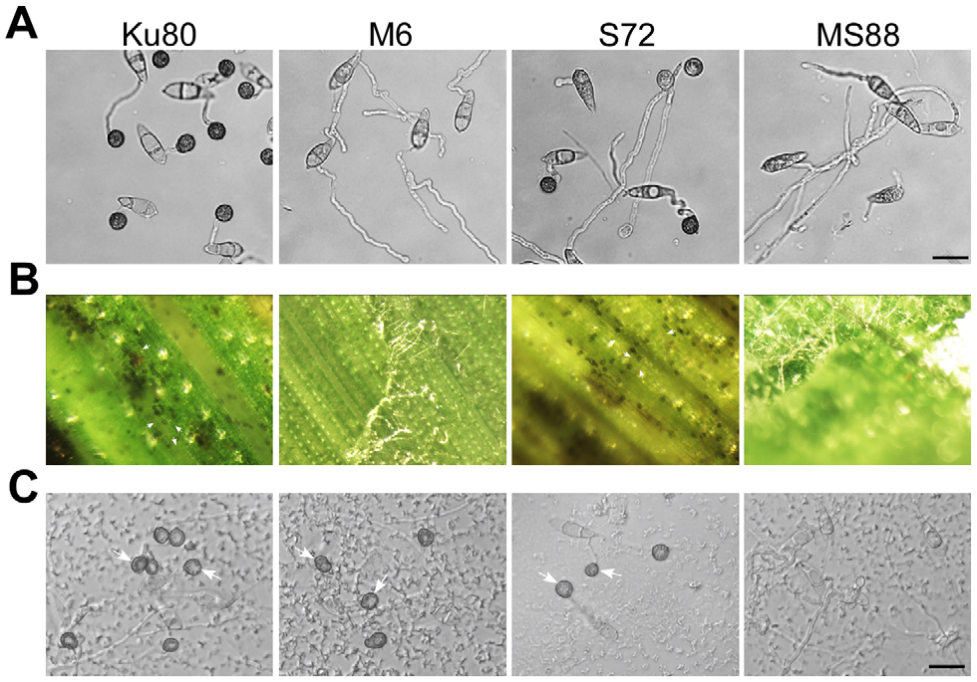
Assays for the effects of different treatments on appressorium formation. Conidia from strains Ku80, M6 (*Momsb2*), S72 (*Mosho1*), and MS88 (*Mosho1 Momsb2*) mutants were incubated on: **A**) the hydrophilic surface in the presence of 10 µM 1,16-hexadecanediol; **B**) de-waxed leaves, and **C**) glass surface coated with the rice leaf wax extract. Representative germlings were photographed after 24 h incubation. Arrows marked appressoria. Bar = 20 µm.

Because cutin monomers, one type of plant surface molecules, are known to trigger appressorium formation in *M. oryzae* [27], we assayed the effects of two cutin monomers on mutants M6, S72, and MS88. In the presence of 10 µM 1, 16-hexadecanediol, over 95% of the wild-type and *Mosho1* mutant germ tubes formed appressoria (Fig. 4A). Under the same conditions, appressorium formation was not induced in the *Momsb2* and *Mosho1 Momsb2* mutants (Fig. 4A). Similar results were obtained with 10 µM cis-9-octadecen-1-ol. Therefore, *MoMSB2* is required for the recognition of these two cutin monomers in *M. oryzae*. Appressorium formation by the *Momsb2* and *Momsb2 Mosho1* mutants on rice or barley leaves is likely induced by plant surface molecules other than cutin monomers that were recognized by a MoMsb2-independent mechanism.

### Leaf surface waxes as chemical cues for appressorium formation

On rice leaves, germ tubes are in close contact with the epicuticular wax layer, which may play a role in surface recognition. To test this hypothesis, epicuticular waxes were removed by dipping rice leaves in hexane for 5 seconds. On de-waxed leaves, Ku80 and the *Mosho1* mutant still efficiently formed melanized appressoria (Fig. 4B). However, the efficiency of appressorium formation by the *Momsb2* and *Mosho1 Momsb2* mutants was significantly reduced (Table S2). The vast majority of mutant germ tubes failed to form appressoria on de-waxed rice leaves (Fig. 4B). Similar results were obtained in appressorium formation assays with intact and de-waxed barley leaves (Fig. S6).

To further prove the role of surface waxes in stimulating appressorium formation in *Momsb2* mutants, crude wax extracts were prepared from rice leaves and used to coat microscope glass slides. In Ku80 and the *Mosho1* or *Momsb2* mutant, about 90% of the germ tubes formed appressoria by 24 h in the presence of the rice leaf wax extract (Fig. 4C). Under the same conditions, less than 32% of the germ tubes formed appressoria in the *Mosho1 Momsb2* double mutant (Fig. 4C), indicating that both *MoSHO1* and *MoMSB2* are important for appressorium formation on hydrophilic surfaces coated with leaf waxes.

### Primary alcohols induce appressorium formation in *M. oryzae*

**Figure 5:**
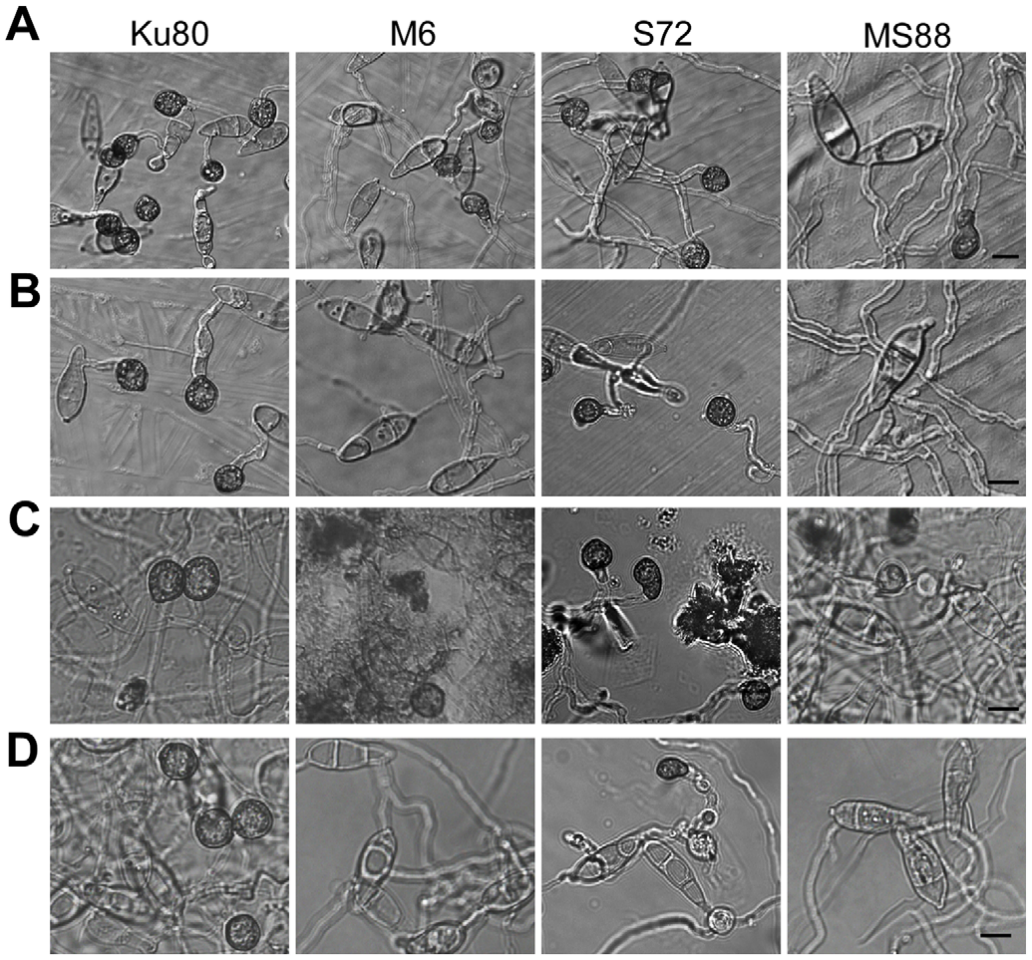
Appressorium formation induced by different waxes or wax components. Conidia from strains Ku80 (WT), M6 (*Momsb2*), *Mosho1* (S72), and MS88 (*Momsb2 Mosho1*) were place over microscope glass slides coated with bee waxes (**A**), paraffin waxes (**B**), C28 primary alcohol 1-Octacosanol (**C**), and C31 alkane hentricacontane (**D**). Bar = 10 µm.

To test whether any rice leaf-specific wax compound is responsible for inducing appressorium formation, we also used bee and paraffin waxes to coat the glass surface. Similar to the rice leaf wax extract, bee wax induced appressorium formation more efficiently in Ku80 and the *Mosho1* or *Momsb2* mutant than in the *Mosho1 Momsb2* double mutants (Fig. 5A). However, paraffin wax could only induce appressorium formation in Ku80 and the *Mosho1* mutant (Table 3; Fig. 5B). In repeated experiments, only bee waxes but not paraffin waxes had stimulatory effects on appressorium formation in the *MoMsb2* and *Mosho1 MoMsb2* mutants. Because coating with paraffin wax changed the surface hydrophobicity (Fig. S7), these results were consistent with the defects of the *Momsb2* and *Mosho1 Momsb2* mutants in recognizing artificial hydrophobic surface. Paraffin wax must lack the components of rice leaf or bee waxes that are recognized by *M. oryzae* as chemical signals for appressorium formation.

**Table 3:** Appressorium formation on glass slides coated with different waxes.

Strain	Leaf waxes^a^	Parafin waxes	Bee waxes	Primary alcohol^c^	Alkane
Ku80	92.3±2.1	91.1±2.3	97.5±0.9	87.1±1.8	85.3±1.2
M6	88.5±1.2	NA^b^	74.2±1.9	70.3±0.7	NA
S72	90.7±0.9	87.4±1.9	96.3±1.3	81.4±1.1	50.3±0.9
MS88	31.1±1.2	NA	49.2±0.7	41.1±2.1	NA

a Percentage of germ tubes formed appressoria. Mean and standard error were calculated from three independent replicates.

b No appressoria were observed.

c The primary alcohol and alkane used in this study were 1-triacontanol (C30) and hentricacontane (C31), respectively.

Plant surface waxes contain primary and secondary alcohols, aldehydes, ketones, alkanes, esters, and long-chain fatty acids [28]. In contrast, paraffin wax mainly consists of long chain alkanes. Because primary alcohols are the major components of surface waxes in grasses, we tested the effects of primary alcohols and alkanes on appressorium formation in *M. oryzae*. On hydrophilic surfaces coated with 1-octacosanol (C28) and 1-triacontanol (C30), the *Mosho1 Momsb2* mutant formed appressoria but less efficiently than Ku80 and the *Mosho1* or *Momsb2* mutant (Fig. 5C). In contrast, the C29 and C31 alkanes (nonacosane and hentricacontane alkanes) induced appressorium formation in the wild-type strain but not in the mutants (Table 3; Fig. 5D). These results indicate that primary alcohols of epicuticular waxes are among the chemical cues recognized by *M. oryzae*.

### *MoMSB2* is important for appressorium penetration

**Figure 6:**
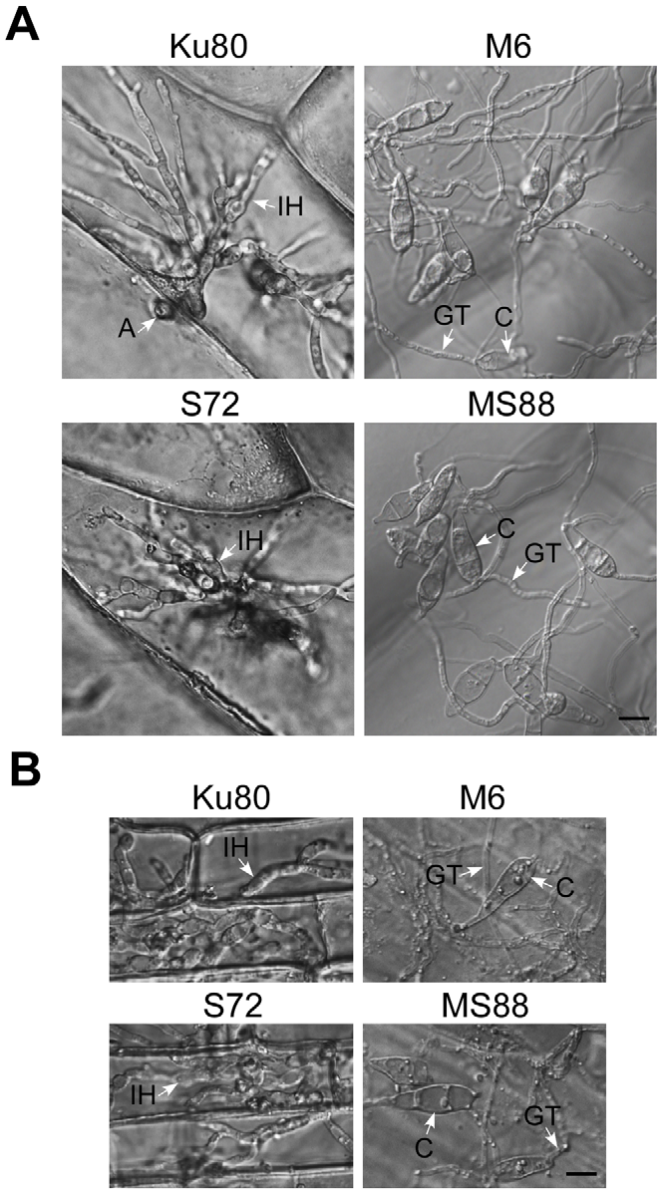
Appressorium penetration assays with onion and rice epidermal cells. **A**. Onion epidermal cells inoculated with Ku80, M6 (*Momsb2*), S72 (*Mosho1*), and MS88 (*Momsb2 Mosho1*) were examined 48 hpi. **B**. Epidermal cells of rice leaf sheaths were inoculated with the same set of strains and examined 48 hpi. A, appressorium; C, conidium; GT, germ tube; IH, infectious hypha. Bar = 10 µm.

In penetration assays with onion epidermises (Fig. 6A), appressoria formed by Ku80 and the *Mosho1* mutant produced invasive hyphae inside plant cells by 48 hpi. Under the same conditions, most (over 99%) conidia from the *Momsb2* and *Mosho1 Momsb2* mutants produced long germ tubes without tip differentiation. Rare appressoria formed by these two mutants failed to penetrate onion epidermal cells. Similar results were obtained in penetration assays with rice leaf sheaths (Fig. 6B). Because the inner surface of rice leaf sheaths lacks epicuticular waxes [29], efficient formation of appressoria by the *Momsb2* and *Mosho1 Momsb2* mutants on rice leaves but not on leaf sheaths further shows that epicuticular waxes are recognized by *M. oryzae* as one of the surface signals. While appressorium formation was not observed in the double mutant, rare appressoria formed by the *Momsb2* mutant failed to penetrate rice leaf sheath cells, indicating that *MoMSB2* is important for appressorium penetration, which may explain why the *Momsb2* mutants formed abundant melanized appressoria but rarely caused lesions on rice leaves.

### MoMsb2 functions upstream from the Pmk1 pathway

**Figure 7:**
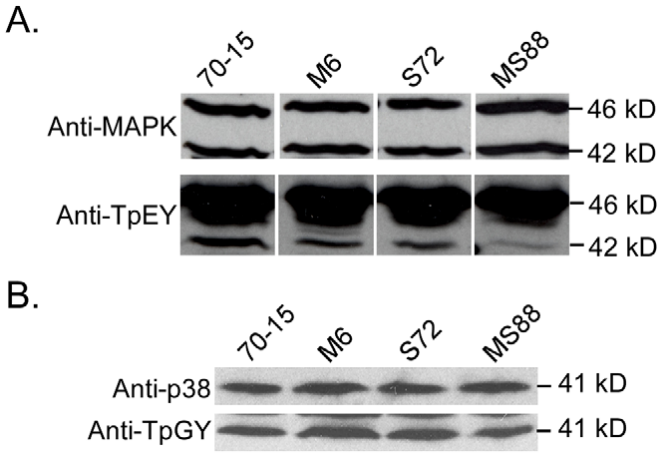
Assays for MAP kinase phosphorylation. Western blots were conducted with proteins isolated from vegetative hyphae of the wild-type (70-15) and the *Momsb2* (M6), *Mosho1* (S72), and *Mosho1 Momsb2* (MS88) mutant strains. **A**. The anti-MAPK and anti-TpEY antibodies detected the expression and phosphorylation levels of Pmk1 (42-kD) and Mps1 (46-kD), respectively. **B**. The 41-kD Osm1 band was detected with both an anti-P38 MAPK antibody and the anti-TpGY antibody.

Although Pmk1 expression was not affected, the activation of Pmk1 as detected with an anti-TpEY antibody was reduced in the *Momsb2* and *Mosho1 Momsb2* mutants (Fig. 7A). In the same western blot analysis, the expression and phosphorylation of Mps1 [30] was normal in these three mutants (Fig. 7A). Osmoregulation is mediated by Osm1, the third MAPK in *M. oryzae* [30]. When detected with an anti-TpGY antibody, the wild type and mutants had similar levels of Osm1 phosphorylation (Fig. 7B), suggesting that *MoMSB2* and *MoSHO1* play only a minor role, if any, in the osmoregulation pathway. Overall, Pmk1 was the only MAPK with a reduced phosphorylation level in *Momsb2* deletion mutants, indicating that *MoMSB2* may function upstream from the Pmk1 MAP kinase.

**Figure 8:**
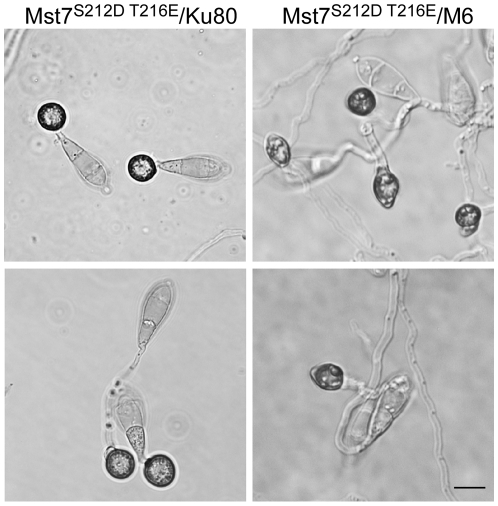
Effects of expressing the dominant active allele of *MST7* on appressorium formation. Conidia from transformants of the wild-type strain 70-15 (WDA2) and the *Momsb2* mutant M6 (WDA12) expressing the *MST7* ^DA^ allele were incubated on hydrophobic (upper) or hydrophilic (lower) surface of Gelbond membranes for 24 h. Bar = 10 µm.

The *MST7* gene encodes a MEK that activates Pmk1. Expressing a dominant active allele of *MST7* induces appressorium formation on hydrophilic surfaces [14]. We introduced this *MST7* ^DA^ allele into the *Momsb2* mutant. In the resulting transformants WDA2 and WDA12 (Table 1), appressorium formation was observed on hydrophilic and hydrophobic surfaces (Fig. 8), indicating that expression of the *MST7* ^DA^ allele induced appressorium formation in the *Momsb2* mutant. Therefore, MoMsb2 may function upstream from the Pmk1 MAPK cascade for appressorium formation.

### Expression and localization of MoMsb2

**Figure 9:**
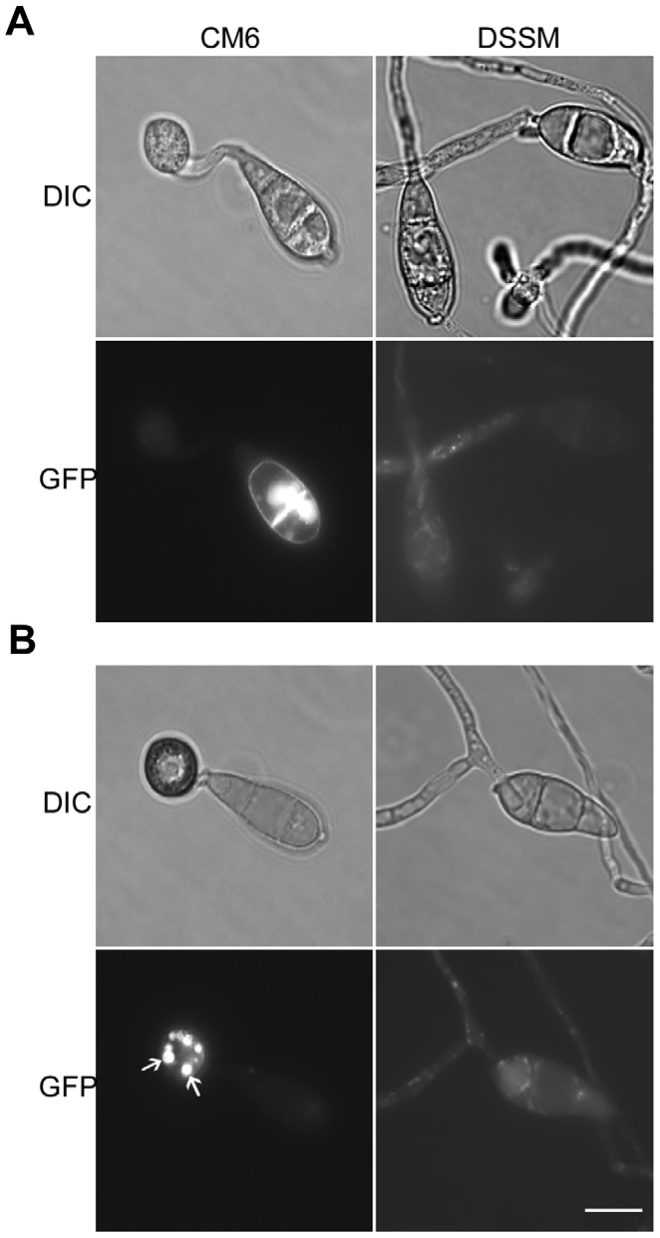
Expression and subcellular localization of MoMsb2-eGFP. **A**. Conidia, germ tubes, and young appressoria of the *MoMSB2*-eGFP (CM6) and *MoMSB2* ^ΔSP^-eGFP (DSSM) transformants were examined by DIC or epifluoresence microscopy. **B**. In mature appressoria (24 h) of transformant CM6, GFP signals localized to small vacuole-like structures (marked with arrows). In transformant DSSM, appressorium formation was not observed and GFP signals localized in the cytoplasm. Bar = 10 µm. The same fields were examined under DIC (left) and epifluoresence microscopy (right).

In transformant CM6 expressing the *MoMSB2*-eGFP fusion construct, GFP signals were detected mainly in vacuole-like structures but also on the cytoplasmic membrane in vegetative hyphae and conidia (Fig. 9; Fig. S8). During conidium germination and appressorium formation, fluorescent signals were detected on the cytoplasmic membrane and in vacuoles that were visible under DIC microscopy (Fig. 9A). Interestingly, germ tubes and young appressoria had no or very weak fluorescent signals. In mature appressoria (24 h), GFP signals mainly localized to vacuole-like structures (Fig. 9B).

The bulk of the mature MoMsb2 protein is predicted to be extracellular. To test the importance of the signal peptide, we generated the *MoMSB2* ^ΔSP^-eGFP allele and introduced it into mutant M6. Transformant DSSM (Table 1) expressing this mutant allele, similar to the original *Momsb2* mutant, was defective in appressorium formation (Fig. 9) and plant infection. Weak GFP signals were detected in vegetative hyphae, conidia, and germ tubes. However, the subcellular localization pattern of GFP signals in transformant DSSM differed from that of transformant CM6. In transformant DSSM, GFP signals mainly localize to the cytoplasm (Fig. 9B). Therefore, the signal peptide is essential for the localization and function of MoMsb2.

### Functional characterization of different domains of MoMsb2

**Figure 10:**
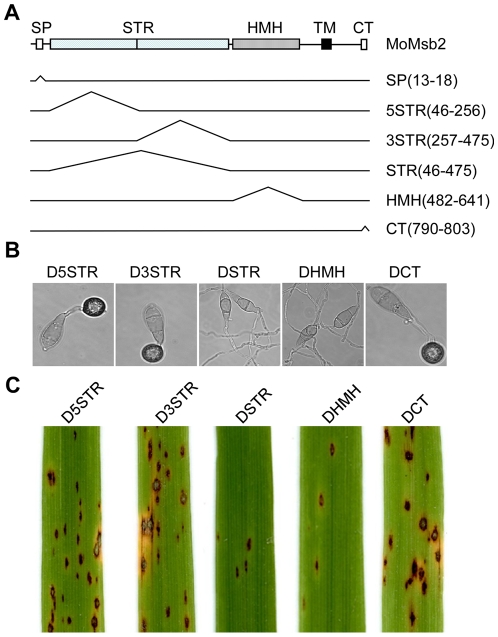
Functional characterization of different domains of the *MoMSB2* gene. **A**. Schematic drawing of different mutant alleles of *MoMSB2*. The numbers in the bracket indicate the amino acid residues deleted in each allele. **B**. Appressorium formation assays with transformants D5STR, D3STR, DSTR, DHMH, and DCT that expressed the *MoMSB2* ^Δ5STR^-, *MoMSB2* ^Δ3STR^-, *MoMSB2* ^ΔSTR^-, *MoMSB2* ^ΔHMH^-, and *MoMSB2* ^ΔCT^-eGFP alleles. Bar = 10 µm. **C**. Rice leaves inoculated with conidia from the same set of strains. Like the original *Momsb2* mutant, transformants DSTR and DHMH were significantly reduced in appressorium formation on hydrophobic surfaces and virulence.

In addition to the signal peptide, MoMsb2 has a STR region, a HMH domain, and a CT domain. We generated mutant alleles of *MoMSB2*-GFP deleted of different regions (Fig. 10A) and transformed them into the *Momsb2* mutant M6. The resulting transformants (Table 1) were confirmed by PCR analysis. Transformants expressing the *MoMSB2* ^ΔHMH^- and *MoMSB2* ^ΔSTR^-eGFP alleles were, similar to the original *Momsb2* mutant, defective in appressorium formation (Fig. 10B) and plant infection (Fig. 10C), indicating that the STR and HMH domain are essential for MoMsb2 function. In contrast, the CT domain was dispensable for appressorium formation (Fig. 10B) and virulence (Fig. 10C). Transformants expressing the *MoMSB2* ^Δ5STR^-, and *MoMSB2* ^Δ3STR^-eGFP alleles were only slightly reduced in appressorium formation and plant infection (Fig. 10B and 10C). Therefore, the N-terminal and C-terminal regions of the Ser- and Thr-rich mucin domain have redundant functions in MoMsb2.

## Discussion

Mucin proteins are characterized by the Ser- and Thr-rich mucin domain and divided into secreted and cell surface (signaling) mucins [31]. Muc1 is the most extensively studied cell surface mucin that affects various cellular functions in mammalian cells, including MAPK signaling. In yeast, *MSB2* was first isolated as a multiple copy suppressor gene of a temperature sensitive allele of *CDC24*. Although it is dispensable for yeast growth under normal conditions, the Msb2 surface mucin interacts with Cdc42 and Sho1 for regulating filamentous growth [22], [32]. One other mucin gene in *S. cerevisiae* is *HKR1* that is not related to the filamentation pathway [33]. In *M. oryzae*, MoMsb2 has structural components conserved in cell surface mucins (Fig. 1). The other mucin-like protein in *M. oryzae* is the chitin-binding protein Cbp1 [34], which shares limited homology (15% identity) with Hkr1 but lacks the mucin and TM domains. Therefore, *MoMSB2* likely is the only cell surface mucin gene in *M. oryzae*.

In *M. oryzae*, *MoMSB2* appears to play a minor role in regulating vegetative growth. The *Momsb2* mutant was slightly reduced in the growth rate but it was normal in conidiation (Table 2). In contract, the *Mosho1* and *Mosho1 Momsb2* mutants were reduced over 5-fold in conidiation (Table 2), suggesting that *MoSHO1* is involved in the regulation of conidium production in *M. oryzae*. However, transformants of *Mosho1* mutants expressing the wild-type allele of *MoSHO1* were increased in conidiation about 3-fold but they still produced fewer conidia than Ku80 (Table 2). These results indicate that ectopic integration of *MoSHO1* did not fully complement the defect of *Mosho1* mutant in conidiation. Nevertheless, the *Mosho1*/*MoSHO1* complemented strain was complemented in the growth rate and appressorium formation (Table 2). Therefore, it is likely that the *Mosho1* mutant was reduced in conidiation due to deletion of *MoSHO1* together with an un-related event during transformation.

Surface hydrophobicity and cutin monomers are two well known surface signals recognized by *M. oryzae* [30]. The *Momsb2* mutant was significantly reduced in appressorium formation on hydrophobic surfaces (Table 2). It also failed to respond to two cutin monomers. These results indicate that MoMsb2 is involved in sensing surface hydrophobicity and cutin monomers. Interestingly, the *msb2* and *sho1* genes were recently shown to be essential for appressorium formation on artificial hydrophobic and plant surfaces in *U. maydis* [24]. However, appressoria formed by *U. maydis* lack the distinct morphological features of *M. oryzae* appressoria. It may be difficult to observe rare appressoria formed by the *sho1 msb2* mutant. The *Momsb2* mutant still formed appressoria efficiently on intact rice or barley leaves, suggesting that *MoMSB2* is not essential for recognizing leaf surface waxes. Therefore, surface hydrophobicity, cutin monomers, and waxes are sensed by different mechanisms in *M. oryzae*. In comparison with the single mutants, the *Mosho1 Momsb2* double mutant had more severe defects in appressorium formation and virulence. The *MoMSB2* and *MoSHO1* genes must have overlapping functions in appressorium development and plant infection. In yeast, the *sho1 msb2* mutant displays more severe defects in filamentous growth than the *msb2* mutant [22]. In *M. oryzae*, MoSho1 may play a role in the recognition of rice leaf waxes.

On glass surfaces coated with bee or rice leaf waxes, 90% of *Momsb2* germ tubes differentiated appressoria, but less than 32% of the germ tubes formed appressoria in the *Mosho1 Momsb2* mutant. We also observed that primary alcohols were more efficient in inducing appressorium formation in the *Momsb2* mutant than in the *Mosho1 Momsb2* double mutant. These results further prove that *MoMSB2* is not important for appressorium formation induced by leaf waxes. The difference between the *Momsb2* and *Mosho1 Momsb2* mutants in the efficiency of wax-induced appressorium formation suggests that *MoSHO1* plays a more important role than *MoMSB2* in recognizing surface waxes as chemical signals for appressorium formation. Coating with waxes changed the surface hydrophobicity, which may be recognized by MoMsb2 and resulted in induced appressorium formation in the *Mosho1* mutant. However, the *Mosho1 Momsb2* mutant still formed a few appressoria on plant leaves or wax-coated glass slides. Additional sensor genes must exist in *M. oryzae* for recognizing wax components. Different genes may be responsible for responding to specific physical or chemical signals in the rice blast fungus.

Bee and rice leaf waxes, but not paraffin wax, induced appressorium formation in the *Momsb2* deletion mutants. Because coating with paraffin wax changed the surface hydrophobicity, these results indicate that physical signals related to hydrophobicity are not sufficient to trigger appressorium development in mutants deleted of *MoMSB2*. Some components of bee and rice leaf waxes must be recognized as chemical signals by the *Momsb2* deletion mutants. Unlike rice leaf waxes comprised of alcohols, aldehydes, ketones, alkanes, and esters [28], paraffin wax mainly consists of long chain alkanes. In further experiments, two C29 and C31 alkanes, nonacosane and hentricacontane, failed to induce appressorium formation in mutant M6 or MS88. In contrast, these mutants formed melanized appressoria on hydrophilic surfaces coated with two primary alcohols 1-octacosanol (C28) and 1-triacontanol (C30). Therefore, primary alcohols but not alkanes in leaf waxes may be responsible for inducing appressorium formation in the *Momsb2* and *Mosho1 Momsb2* mutants. One major component of leaf epicuticular waxes in grass species is primary alcohols [35]. Other plant pathogenic fungi may also recognize primary alcohols for regulating infection-related morphogenesis. Because grapes (one of the native environments for the budding yeast) also are covered with waxes, it is possible that waxes play a role in inducing filamentous growth in *S. cerevisiae*.

Deletion of *MoMSB2* resulted in defects in appressorium formation on artificial surfaces and plant penetration, two processes regulated by Pmk1 [36]. Although its expression was not affected, the phosphorylation of Pmk1 was reduced in the *MoMsb2* and *Mosho1 Momsb2* mutants. To further prove that MoMsb2 and MoSho1 function upstream from the Pmk1 cascade, the dominant active allele of *MST7* [14] was transformed into the *Momsb2* mutant. The resulting transformants formed appressoria on hydrophilic surfaces (Fig. 8). Overall, these results further confirm that many components of the yeast filamentation MAPK pathway are involved in the regulation of infection-related morphogenesis in *M. oryzae*. In *U. maydis*, Msb2 and Sho1 also function upstream a MAPK pathway and are important for plant infection [24]. In nature, filamentation may be important for the budding yeast to colonize the substrates, such as grapes.

Domain deletion analysis with *MoMSB2* indicates that the signal peptide is essential for its function. Interestingly, the *cbp1* and *Momsb2* mutants had similar defects in appressorium formation although the chitin-binding protein Cbp1 protein lacks the transmembrane domain. Both MoMsb2 and Cbp1 are predicted to be heavily glycosylated according to analyses with the (http://cbs.dtu.dk/services/NetNGlyc/) and (http://ogpet.utep.edu/OGPET/). It will be important to generate and characterize the *Momsb2 cbp1* double mutant and determine the relationship between these two genes. MoMsb2 and Cbp1 may be functionally related by forming a surface complex for recognizing different extracellular signals to activate the Pmk1 pathway.

Based on GFP signals observed in transformant CM6, *MoMSB2* is constitutively expressed. The MoMsb2-eGFP protein may localize to the cytoplasmic membrane in its inactive form. Localization to the vacuoles or vacuole-like structures may be related to the internalization of the fusion proteins. In yeast, the cleavage of Msb2 at the cleavage domain (located upstream from the TM domain) is essential for its activity [37]. MoMsb2 has the sequence element adjacent to the TM domain that is similar to the Msb2 cleavage domain. The activation of MoMsb2 may involve protein cleavage at this site and result in its dissociation from the cytoplasmic membrane and diffusion into cell wall and extracellular space, which may explain the absence of GFP signals in germ tubes and young appressoria of transformant CM6. It is possible that only the cleaved MoMsb2 (the active form) interacts with other extracellular proteins (such as Cbp1) to sense various physical and chemical signals for appressorium formation. Therefore, characterizing the role of protein cleavage in the activation and localization of MoMsb2 may provide critical information about surface recognition mechanisms in *M. oryzae*.

*PTH11* encodes a putative GPCR that is involved in recognifzing surface hydrophobicity in *M. oryzae* [19]. In the *pth11* mutant, about 10–15% of the germ tubes still form appressoria on hydrophobic surfaces [19], which is approximately five times higher than that of the *Momsb2* mutant. In addition, the *pth11* mutant, unlike the *Momsb2* mutant, still responds to cutin monomers for appressorium formation. *PTH11* likely functions upstream from cAMP signaling [19] but its role in the activation of the *PMK1* MAPK pathway has not been studied. *PTH11* and *MoMSB2* may be functionally related in recognizing different chemical and physical signals present on the rice leaf surface. It will be important to determine their relationship in the activation of the cAMP-PKA and *PMK1* MAPK pathways for regulating appressorium formation and penetration.

## Materials and Methods

### Strains and culture conditions

All the wild-type and mutant strains of *M. oryzae* (Table 1) were cultured on oatmeal agar plates (OA) at 25°C. Culture preservation, genetic crosses, transformation, and measurements of conidiation and growth rate were performed as described [38], [39], [40]. For nucleic acid and protein isolation, vegetative hyphae were harvested from two-day-old liquid CM cultures [41]. Lesions formed on 5-cm rice leaf tip segments were counted as described [42], [43].

### Deletion of *MoSHO1* and *MoMSB2*

Approximately 0.8-kb upstream and downstream flanking sequences of *MoMSB2* were amplified with primers 11F/12R and 13F/14R (Table S1) and ligated with the *hph* cassette. The *MSB2* gene replacement construct was amplified with primers 11F and 14R and transformed into Ku80 [26]. To delete the *MoSHO1* gene, a DNA fragment containing the entire *MoSHO1* and its 710-bp upstream and 329-bp downstream flanking sequences were amplified with primers 1F and 2R, and cloned into pGEM-T easy vector (Promega, Madison, WI). The *MoSHO1* gene replacement construct pLL9 (Fig. S2) was generated by replacing a 355-bp *Bam*HI/*Bsi*WI fragment of *MoSHO1* with the *hph* cassette amplified from pCB1003 with primers HPH5F and HPH4R.

To generate the *Mosho1 MoMsb2* double mutant, the *MoMSB2* replacement construct was generated with a bleomycin-resistant cassette and transformed into the *Mosho1* deletion mutant S72. Transformants resistant to both hygromycin and bleomycin were screened by PCR. For complementation assays, the *MoMSB2* gene was cloned into pYP1 that was generated by replacing the *hph* gene in pDL1 [44] with the bleomycin-resistance gene. The resulting construct pXY130 was transformed into the *Momsb2* mutant M6. The *MoSHO1* complementation vector pKNSHO1 was transformed into mutant S72 or co-transformed with pXY130 into the double mutant MS88.

### Complementation of yeast *sho1* and *msb2* mutants

The *MoSHO1* ORF was amplified with primer Sho1F/Sho1R and cloned into pYES2 as pMoSHO1. The same procedure was used to generate plasmid pMoMSB2. The resulting constructs were transformed into the *sho1* mutant obtained from Open Biosystems (Huntsville, AL) and the *msb2* mutant [45] with the alkali-cation yeast transformation kit (MP Biomedicals, Solon, OH). Ura3^+^ transformants were assayed for invasive growth and sensitivity on YPD and YPGal plates with 1.5 M sorbitol as described [32], [46].

### Appressorium formation and penetration assays

Conidia were harvested from 10-day-old OA cultures, resuspended to 5×10^4^ conidia/ml in sterile water, and used for appressorium formation assays with glass cover slips (Fisher Scientific, Pittsburgh, PA) or Gelbond membranes (FMC, Philadelphia, PA) as described [40], [47]. Penetration assays were conducted with onion epidermal cells and rice leaf sheaths [4], [48].

### Infection assays with detached barley leaves

Conidia were harvested from 10-day-old OA cultures and resuspended to 5×10^5^ conidia/ml in 0.25% gelatin. Two-week-old seedlings of rice cultivars Nipponbare and CO-39 were used for spray or injection infection assays as described [49]. Eight-day-old seedlings of barley cultivar Golden Promise were used for spray or drop inoculation assays as described [50]. Lesion formation was examined 5–7 days post-inoculation (dpi). For assaying changes in virulence, lesions formed on 5-cm leaf segments were counted as described [38], [51].

### Wax extraction and treatments

Rice leaves of two-week-old seedlings were dipped in hexane for 20 s. Epicuticular waxes dissolved in hexane were recovered by evaporation under a nitrogen stream [52]. For coating glass surface, 10 mg of the rice leaf surface wax was dissolved in 3 ml chloroform. Aliquot of 50 µL were dropped onto microscope glass slides (Gold Seal, Portsmouth, NH). Bee wax (Stakich Inc., Bloomfield Hills, MI) and paraffin wax (Oak Sales, San Diego, CA) were directly applied on to glass surface. Drops of 25 µl conidium suspensions were placed on wax-coated areas and assayed for appressorium formation. The C28 (1-octacosanol, C28H58O) and C30 (1-triacontanol, C30H62O) primary alcohols and C29 (nonacosane, C29H60) and C31 (hentricacontane, C31H64) alkanes (Sigma) were dissolved to 4 mg/ml in chloroform.

### Scanning electron microscopy (SEM) examination

Barley and rice leaves inoculated with the wild-type and mutant conidia were sampled at 24 hpi and immediately frozen in liquid nitrogen. After being sputter-coated with gold in the presence of argon in a Hexland CT-1000 cryo-system (Gatan, Pleasanton, CA), leaf samples were examined at −140°C in a JEOL JSM-840 scanning electron microscope for germ tube growth and appressorium formation [49].

### Construction of *MoMSB2*-eGFP and *MoSHO1*-eGFP

A 4.2-kb fragment of the *MoMSB2* gene was amplified with primers MGFPF and MGFPR (Table S1) and co-transformed with *Xho*I-digested pYP1 into XK1-25 [42]. For *MoSHO1*, a 2.8-kb fragment was amplified with primers SGFPF and SGFPR and co-transformed into XK1-25 with *Xho*I-digested pDL2 [44]. Plasmids pXY130 and pXY122 containing the *MoMSB2*-eGFP and *MoSHO1*-eGFP fusion constructs, respectively, were recovered from Trp+ yeast transformants and transformed into protoplasts of 70-15. For complementation assays, the *MoMSB2*-eGFP and *MoSHO1*-eGFP constructs were transformed into the *Momsb2* mutant M6 and the *Mosho1* mutant S72, respectively. Zeocin-resistant transformants were examined for GFP signals in conidia, appressoria, and infectious hyphae as described [42], [53].

### Domain deletion analyses of *MoMSB2*

To delete the signal peptide, PCR products amplified with primers MGFPF/ΔSSR1 and ΔSSF2/MGFPR were cotransformed with *Xho*I-digested pYP1 into *S. cerevisiae* strain XK1-25. Plasmid pXY148 was recovered from yeast Trp+ transformants and confirmed by sequencing analysis to contain the *MoMSB2* ^ΔSP^ construct. The same yeast gap repair approach [44] was used to generate the *MoMSB2* ^ΔHMH^, *MoMSB2* ^Δ**CT**^, *MoMSB2* ^Δ5STR^, *MoMSB2* ^Δ3STR^, and *MoMSB2* ^ΔSTR^ alleles. PCR primers for generating these mutant alleles were listed in supplemental table 1. All domain deletion constructs were transformed into the *Momsb2* mutant M6.

### qRT-PCR analysis

RNA samples were isolated from mycelia from two-day-old liquid CM cultures and 24 h appressoria with the Trizol Reagent (Invitrogen, CA). First-strand cDNA was synthesized with the AccuScript 1st strand cDNA synthesis kit (Stratagene, La Jolla, CA). RT-PCR was performed with the Stratagene Gene MX 3000 PM using the RT2 Real-TimeTM SYBR Green/ROX PCR master mix (SABiosciences, MD). Primer pairs Msb2QF/Msb2QR and AQF/AQR were used to amplify the *MoMSB2* and actin (MGG_03982) genes, respectively. The relative quantification of each transcript was calculated by the 2-^ΔΔ^CT method [54] with the actin gene as the internal control.

### Protein isolation and western blot analysis

Vegetative hyphae were harvested from 2-day-old CM cultures and used for protein extraction as described [42], [55]. Total proteins (approximately 20 mg) were separated on a 12.5% SDS-PAGE gel and transferred to nitrocellulose membranes for western blot analysis [56]. TEY- and TGY-specific phosphorylations of MAP kinases were detected with the PhophoPlus p44/42 and p38 MAP kinase antibody kits (Cell Signaling Technology, Danvers, MA) following the manufacturer's instructions.

### GenBank accession numbers

Sequence data for genes described in this article can be found in the GenBank under the following accession numbers: *MSB2* (NP_011528), *SHO1* (NP_011043), *MoMSB2* (MG06033), *MoSHO1* (MG09125), *Aspergillus nidulans AnMSB2* (ANID_07041), *A. nidulans AnSHO1* (ANID_07698), *Neurospora crassa NcMSB2* (NCU04373), *N. crassa NcSHO1* (NCU08067), *Candida albicans CaMSB2* (XP_722538), *C. albicans CaSHO1* (CAC81238).

## Supporting Information

Figure S1 Alignment of MoMsb2 (A) and MoSho1 (B) with corresponding orthologs from *Neurospora crassa* (Nc), *Aspergillus nidulans* (An), *Candida albicans* (Ca), and *Saccharomyces cerevisiae*. Identical and similar residues were shaded in black and gray, respectively. STR, serine/threonine rich region; HMH, Hkr1-Msb2 homology domain; TM, transmembrane domain; CT, cytoplasmic tail; SH3, and Src homology 3 domain. The STR region was not well conserved in Msb2 orthologs.
(0.46 MB RTF)

Figure S2 Nucleotide sequence of the *MoMSB2* promoter region. Two PRE-like sequences were underlined. Two putative TCS elements were shaded in gray.
(0.02 MB DOC)

Figure S3 The *Momsb2* and *Mosho1* deletion mutants. A. The MoSHO1 gene replacement event and Southern blot analysis. Genomic DNA samples of 70-15 (WT), S72 (*Mosho1*), Ect7, and Ect12 (ectopic) were digested with BclI. The blot on the left was hybridized with probe 1 amplified with 1F and 6R. On the right was the same blot stripped and re-hybridized with probe 2 amplified with 5F and 7R. B. The *MoMSB2* gene replacement events and Southern blot analysis of the *Momsb2* (M6) and *Mosho1 Momsb2* (MS88) mutants. When hybridized with a fragment of the *MoMSB2* gene (probe 3), the wild-type 5.7-kb NcoI band was absent in mutants M6 and MS88. When hybridized with a downstream fragment of *MoMSB2* (probe 4), mutants M6 and MS88 lacked the wild-type 5.7-kb band but had the expected 3.8-kb and 4.3-kb bands, respectively.
(0.70 MB TIF)

Figure S4 Lesions formed by the *Momsb2* and *Mosho1 Momsb2* mutants on rice leaves. A representative leaf tips sprayed with 5×10^4^ conidia/ml conidia from the *Momsb2* and *Mosho1 Momsb2* mutants. Typical leaves were photographed 7 dpi. B. Close view of lesions caused by the *Momsb2* and *Mosho1 Momsb2* mutants.
(1.18 MB TIF)

Figure S5 Appressorium formation and plant infection assays with the complemented transformants. A. Melanized appressoria formed by transformants CM6 (*Momsb2/MoMSB2*) and CMS74 (*Mosho1 Momsb2/MoSHO1 MoMSB2*) on hydrophobic surfaces. Bar = 10 µm. B. Blast lesions formed on rice leaves inoculated with CM6 and CMS74.
(0.57 MB TIF)

Figure S6 Barley leaves were inoculated with strains Ku80, M6 (*Momsb2*), and MS88 (*Momsb2 Mosho1*) and examined under SEM. The mutants formed appressoria on intact barley leaves (upper panels) but not on de-waxed leaves. Bar = 10 µm.
(0.50 MB TIF)

Figure S7 Surface hydrophobicity of waxed microscope glass slides. Drops of 20 µl of 0.02% bromophenol blue (BPB) in distilled water were placed onto the surface of glass slides that were untreated or coated with bee waxes.
(0.85 MB TIF)

Figure S8 Time course assays for the expression and localization of MoMsb2-eGFP during appressorium formation. Representative images were presented for each time point (labeled on top). Bar = 10 µm. The same fields were examined under DIC (left) and epifluoresence microscopy (right).
(0.28 MB TIF)

Table S1 PCR primers used in this study.
(0.05 MB DOC)

Table S2 Appressorium formation on intact and de-waxed rice leaves.
(0.03 MB DOC)
