# Supplementary material for: Multiple Plant Surface Signals are Sensed by Different Mechanisms in the Rice Blast Fungus for Appressorium Formation
Source: PLoS Pathog. 2011 Jan 20;7(1):e1001261. doi: 10.1371/journal.ppat.1001261 (PMC3024261; doi:10.1371/journal.ppat.1001261)
Supplement: Figure S1 — Alignment of MoMsb2 (A) and MoSho1 (B) with corresponding orthologs from Neurospora crassa (Nc), Aspergillus nidulans (An), Candida albicans (Ca), and Saccharomyces cerevisiae. Identical and similar residues were shaded in black and gray, respectively. STR, serine/threonine rich region; HMH, Hkr1-Msb2 homology domain; TM, transmembrane domain; CT, cytoplasmic tail; SH3, and Src homology 3 domain. The STR region was not well conserved in Msb2 orthologs. (0.46 MB RTF) [file ppat.1001261.s001.rtf]

A
MoMsb2    1 ------------------------------------------------------------------------------------------------------------------------
NcMsb2    1 ------------------------------------------------------------------------------------------------------------------------
AnMsb2    1 ------------------------------------------------------------------------------------------------------------------------
ScMsb2    1 MQFPFACLLSTLVISGSLARASPFDFIFGNGTQQAQSQSESQGQVSFTNEASQDSSTTSLVTAYSQGVHSHQSATIVSATISSLPSTWYDASSTSQTSVSYASQESDYAVNQNSWSASTN
CaMsb2    1 -------------MLANVKLNLVTALYVLSYVSVVNAYQQENEITPADNIDKRAGAIGNFFRDFTNSIFGNDNSEVNQPSTNGATSTGHFFGPSIPSTSTHQQTPGETSNNVNTKSSSQN
                                           SP                  STR
MoMsb2    1 -----MHNFSKLAVAFVAAASFASAEPETKAKVERPIIYFPRHIKRQFANTTTPASEASSSTSRPPPIPVPETSSFSSSASS--------------------------------------
NcMsb2    1 -----MRTVSVVAAALLAASYVAGEHVG----PTKPKYYFPKHVKRQYANATITSNDAPISTIDTSSDALSQTTKRETTSNDQS---------------------ESLSDIPFVKSTSSR
AnMsb2    1 ----------MVSQTALIAAALSLAGLELVAAQQPNAHHEHKQLKRQLFGSSDQSNNLGWLESFLSSNSDSGTTSSTTTSQN--------------------------------------
ScMsb2  121 QLPSTSTTSYYAPTFSTSADFAASSVNAASDVSTASVPIDTSANSIPFTTTSNIETTTSAPLTSDTPLISTSTMSAADNVFSSAN-----------------PISASLTTTDSSESFDQT
CaMsb2  108 QSPSTSPTSTVAAAAATSSSPVASTRPASTSEQKQQEETTARQSTSPATTATTSNTPPSPSTSKETPTSNTAQTSSANNNQQSSNTAAPSTSVIQPSTSEVHVQSQQTSTTPNTPTSSPN
              STR
MoMsb2   78 --SSAQELTASRQPTSIDEFFSTLSDALTTDSTPFSQRPATSGAGRSSATGDVTPIIVPSSASPPSTAVKPGSVSALTTSQN-----------------STSAATSESVTSPGSTSGPAG
NcMsb2   91 FLTSSEPQSESESDTSRVTTVVIASTVYVSPSQPTTADLSTAGTAAPSAPAVDTDFVSSTDSTAGSSDSTSQGTTGLSSSATDNSSTDLLGFPTGISSGSTTESSLSDSTTGGTSSGATS
AnMsb2   73 --SGVTAASSKDSDPDVVIVPITLSVDENGKTHTITGTANTGVATGAASTTTKQSTVAEVTASATTESQPTATSTSSSTREDTDLSGILGSLLGGSSSSDSSASTTQTGISRAATDSTIS
ScMsb2  224 STAGAIPVQSSADFSSSSEILVQSSADFSSPSSPTTTDISLSAAPLQTSESSSFTTASAALPVSSTDVDGSSASPVVSMSAAGQIASSSSTDNPTMSETFSLTSTEVDGSDVSSTVSALL
CaMsb2  228 TPTTSEAAPTTSAAPTTSEAPVTPSTSEVVPNTPTTSEAPNTPTTSEAPVTPSTSEVVPNTPTTSKAPNTPTTSEAPATPTTSEAPNTPTTSEAPVTPTTSEVVPTTSTQGDAVSTSSTS
              STR
MoMsb2  179 TPESSSASDFTSAVATSR---------------ASTATSNTGLIPETTILPTTATSNTGLIPETTILPTTAS----LST-----------------------------------------
NcMsb2  211 SPESTPGTSLGSTTDSSTSSPVTATDRVDSSASASASASASTSASDNTILPSSGANVTSSFASTTDAVSSSSSVTKTAS-----------------------------------------
AnMsb2  191 ETETTDSSTSPSSTSSSGGLLESLLGGSDSSASTSTSSTTSSSTSTSDGLLGLNLNLNGDSTSSTSTTTGSSPSATSTS-----------------------------------------
ScMsb2  344 SAPFLQTSTSNSFSIVSPSVSFVPSQSSSDVASSSTANVVSSSFSDIPPQTSTSGSVVSVAQSASALAFQSSTEVYGASASSTMSSLLSTTS----------------------------
CaMsb2  348 VTEQTTLTSSTQLPPTTASTTQTSTPEASDSPKPSSTSIETPSTSTFEQDPTTTSSVGTPSSEQPQPTTTSESAVTSNSPTQESTSLVEPTTSSLESSNTPTPNPSTSEAQPSTSASQAP
              STR
MoMsb2  239 ----------------------------------------AESAVTPSITSSASSSGILIAPTGVVTPTS----------------------------SSSTEDPVFDGIGTLISSIVSS
NcMsb2  290 ----------------------------------------SSSPTTDSASSSASVTGSVTSPGSIANTTSSAISASSSSSVVDILSFLNPGFGGASSSASATDSTTVLGSVANTTSAAIS
AnMsb2  270 ----------------------------------------SSNGLLENLLGDSTSTSTSTSTADSSSATS-----------------------------TSSSGGLLDGLLGGDSTSSSG
ScMsb2  436 ---------------------------------------LQSTTLDSSSLASSSASSSDLTDYGVSSTASIPLLSASEQASTSSSFSVVSPSVSFVPSQSSSDVASTSAPSVVSSSFSYT
CaMsb2  468 PDTTSSAPAPELSSSNADFSNSVLHSSETTSLVNPTDSQIDSSSTTDAVSQATTEPTSENTPTAASSVTANDINSAQSSAPTSNADAETASSPVSEQSLATGSQTSLDTTAGASSTASEA
              STR
MoMsb2  291 VSTVLQPN------------------------------GTAPVTTTPN------TSVDVATTPVDIASTTASDTLSPTTAVVSTTGPVTSVQTLPPVST---------------------
NcMsb2  370 ATDVASSS------------------------------ASATDSTTALGSVANTTSAAISATDVARSSASATDVASSSAASTDASGSLTSASALPTVSSGVISNTTSPDSSSSLQVLTPP
AnMsb2  321 TTTATSPS------------------------------ATSTSGGLLDGLFGTDSTSSSATTSVTTPTSTSGGLLGGLFGTDSSSSASTAVIPSSTSTS---------------------
Msb2    517 SLQAGGSS-----------------------------MTNPSSSTIVYSSSTGSSEESAASTASATLSGSSSTYMAGNLQSQPPSTSSLLSESQATSTSAVLASSSVSTTSPYTTAGGAS
CaMsb2  588 TAENLSTFGTDGSSDASQTIAETTSNSPDQSVVTPSASASPDVSTLPTGSESGTSLVSGSETSIDTNTVASGSTVIPESSNIPTQSPSQSVVSSDAAASNVSTGSATTDSLAGSETGVQP
              STR
MoMsb2  354 ------------------------------------------------------------------------------------------------------------------------
NcMsb2  460 TTTVEPISTANATTVETASTVTATDGSASTGWLPPIIVLPSIRTSSTSAADVANSTAPALVSGTGRATNSTIG--TATASEIIATEDPLITSSSSSGILLAPTGVVNETTSASTNLIESL
AnMsb2  390 ------------------------------------------------------------------------------------------------------------------------
ScMsb2  608 TEASSLISSTSAETSQVSYSQSTTALQTSSFASSSTTEGSETSSQGFSTSSVLVQMPSSISSEFSPSQTTTQMNSASSSSQYTISSTGILSQVSDTSVSYTTSSSSVSQVSDTPVSYTTS
CaMsb2  708 ISSSATGTSEPVFSSEYNSSEGTTSLVVPTNSELSSTVTGSSETAATAINSESVLTGSSDTAATVTGSESILTGNTETSATAIASESTLTGSTTGATDSAATTIASESVLTGTSDASATV
              STR
MoMsb2  354 -------------PTANGTVTSPPVDSQTTVLPTTTPGLSSDTIVTSPGVTANSTQVPTTVP----------------------------------------------------------
NcMsb2  578 ASNVASLVGSILIPTGNGTATEPTAPVTTSDSVTIPSATEPYASGSGSGVITPSASLTTSQPKPPMM----------------------------------SSGIVSIPIGNSTVSSTKA
AnMsb2  390 ----------------SGGFLDSLFGTDSSTPTPLPGASSTSGGLIGNILPTISVSVPESTPTG--------------------------------------------------------
ScMsb2  728 SSSVSQVSDTPVSYTTSSSSVSQVSDTPVSYTTSSSSVSQVSDTPVSYTTSSSSVSQVSDTSVPSTSS---------------------------------RSSVSQVSDTPVPSTSSRS
CaMsb2  828 IPSESALTGSTTTPIASESVLTGTTSADVSGATTIGSESIFTGTTESTGTPLPTASGTESLDTTVATGTSVSEQSGVETALSTQPTTGTEATVTSGVSQSEQTGTSAVTGVTESSEQIQS
              STR
MoMsb2  403 ---------------------TTIPTTQPPVTEPTITPTVLPPSPNNTVPSNTTTQLPPTQAPTLTQLPTTTTSPALTTPATTPSVAPTS------------------------------
NcMsb2  664 VESGPTTTGIPGSSVPVIPGNSSIPVTSMSDTTVVLPPVTLINSTTVAANSSTIIPVIVTSASAESSVPVSSLEPVTTTQVLIPPPVPTT------------------------------
AnMsb2  438 -------------------SSDAVASSTPTLLPPDLTSILIPSSSSGATSVPVIPQVPTSSVAATPTPSVGISVPSSSSVSVQPAPTTTS------------------------------
ScMsb2  815 SVSQTSSSLQPTTTSSQRFTISTHGALSESSSVSQQASEITSSINATASEYHSIQTTAATQSTTLSFTDANSSSASAPLEVATSTPTPSSKASSLLLTPSTSSLSQVATNTNVQTSLTTE
CaMsb2  948 GATTPATTTASDATAVTNASEASAESQATTTAASEATTGSQVTTAASEAATESQATTAASEAATESQATTAASEAATESQATTPASEPATGSQVTSEVTPATVPSSVTAADTAATSIITS
              STR                                                     HMH
MoMsb2  472 ----------------ATSSANSNDDWLPTTIIVQAPLPSTTGSSTNAPSSAPTVLPSDLPKIINPSDDITEPLGPDMMEIQVAFKFALNYRFITNENPNAGAQIFEYLPKSLKYMEGLT
NcMsb2  754 -TINYPPVTQYT--PTATVSNMPPGYNSPTTILVDQPPPAQTHTVSFVQQTTATALPTDLPKAIAP-DDTTAPKPEDNVFIQIGFNYGFNYPFVAKNN-NAAAQIFRLLPEALAFASSIE
AnMsb2  509 -TASSEDSTTTVGTTTTTTVEAEPTDWVPTSIIIEPSPTETETSSEETTTVAPTQLPGSISPAGG-----VPDAPEGSTLIQLGFTRELRYSFVATHS-LSSSQIFLYIPQGLMYALEEA
ScMsb2  935 STTVLEPSTTNSSSTFSLVTSSDNNWWIPTELITQAPEAASTASSTVGGTQTMTLPHAIAAATQVP-------EPEGYTLITIGFKKALNYEFVVSEP-KSSAQIFGYLPEALNTPFKNV
CaMsb2 1068 PPASAEPSSEVTAVAPSAATTSSTKNWLPSSLVIAETPSSNASKSTESIVQASATGASTSGLPRAITPETTTTPGFDYQVITVGFKSALNYPFVVENS-ISSAQIFQYLPRVLKYPFNGD
                                                HMH
MoMsb2  576 EEQKKRLQVLRVVPLNTEQQLGYVTSVAIATWPKAFFPQLRLDVKTPFSQFYQNTSNGMLAHNLTMLVNPAIDILPGATLDGKPAG--AGSGTGGNGSNGP--NDVFNND----------
NcMsb2  869 S---HRVRVTKLMPMNTVNTLGYWTTLAVVSYPQAYVESLRLDVKIASSPLYNNPTP--LVYNLTMQINPAIDIILGSTLIGDGSGGDSGSNPSNAGNNGN--ADPFTNN----------
AnMsb2  622 S---SDIAMFAISPYDSEATTGYIATVAQAYIPTEKVDVLRKLLHNPISRLYDQPSE--SVKTLMSMIDPSIPLLVGEYDDSSFGGSSGSGDGSGSDGDGDDSNDTYDDS----------
ScMsb2 1047 FT---NITVLQIVPLQ-DDSLNYLVSVAEVYFPTAEIEELSNLITNSSSAFYTDGMG--TAKSMAAMVDSSIPLTGLLHDSNSNSGGSSDGSSSSNSNSGSSGSGSNSNSGVSSSSGNSY
CaMsb2 1187 KS-LQNVSVRRLIPYTASN-IDYTITVAEVYFPKDSVKALGSFITTPGSAIYRNPDS--VLQALASLIDSRIPLTGLVTDDQQVSGSSSDSNPSTN---------SYGSM----------
                                                      TM
MoMsb2  682 ------------NNSTNQSATQRGTVAGIAFGAVSLAAAYGAAMFIVARRYKKKRQAHRRSSSVAT-PSEMRQSG--SPALMGGALLSRDFTHYGGVMG--------PAGGRESHGSNGS
NcMsb2  972 ------------NN-GNQTSQQRGTTAGIVGGAVAVAAAYGAAMFVVARRYKRKKQAHRRASSLGGSPSDMQQMGGGSPALMGGALLSRDFTGYGGVAGGAAAAGAVAPGGRDSHGSGRS
AnMsb2  727 ------------TAGASSSGSTKASSVGIGVGVVAGAAAYGAGMFWVARRYRKKRQLHRRTSSTADQMSDRGGS-------------------------------IFAGGGRLSRGSG--
ScMsb2 1161 QDAGTLEYSSKSNSNVSTSSKSKKKIIGLVIGVVVGGCLYILFMIFAFKYIIRRRIQSQEIIKNPEISSISSSEFGGEKNYNNEKRMSVQESITQSMRIQNWMDDSYYGHGLTNNDSTPT
CaMsb2 1284 ------------DIVSNTKVADKGRIAGITIGAAAGCGLYMTLMVLLFRKFRKSNKALELPITDSESNLGFSDEDSSMLESSSGFSAIFSRINHGGVLTG-----DPNGGGDDMMMMNNN
                                 CT
MoMsb2  779 GRSAGNSARTAGISAPVAQENSLGWN--
NcMsb2 1079 G--MGLSSRTAYISAPVAAENSLGWN--
AnMsb2  802 ------SQRTQMISAPVMAENSLGWN--
ScMsb2 1281 R--HNTSSSIPKISRPIASQNSLGWNEV
CaMsb2 1387 N----NNLRPNNISEPVQASNSLGWYH-


B
                                                               TM                              TM                TM 
MoSho1    1 --------MEHSRGQYGGGRKGMSLGNVIGDPFALATISIAGLAWLIAFIASIVAQIQTTQG--------FPTYTWWTVVFYFFLIPGVFVVVASDTIQTYHVALVGYMACGLVLTTSSV
NcSho1    1 --------MEHGRNSYR--RKGIDMGNIIGDPFALATTSIATLSWIIILFGSIFGFRDQNDGSNGAPVIVWPTYSWFTLVFNFFLILGIFIVIASDSAQTYHVAIVGYLAVGLVGSTSSI
AnSho1    1 -------------------MAALRASNLLGDPFALATSSIAMLGWLIAFIASIAADVQDP----------YPSFQWWAIAYSFCCNVGVIVVFLTDTGLTYGVAVVGYLAASLVMNSISA
ScSho1    1 MSISSKIRPTPRKPSRMATDHSFKMKKFYADPFAISSISLAIVSWVIAIGGSISSASTNES---------FPRFTWWGIVYQFLIICSLMLFYCFDLVDHYRIFITTSIAVAFVYNTNSA
CaSho1    1 --------------------MGFSLSNFTSDPFAISTVSFGIMAWVVAIAG--AASSKQEN---------FPHFSWWGISYQIVIILIIFVLYANNNIELYKFTLVGLVSIAFIYTTNST

TM
MoSho1  105 NGLVYS-TNGAKEAAAAGFILLSMVTIVWIFYFGSAPSAMPRAYLDSFALSKESTS----NNRQTMTGGGYG--------IGRPETSTSVQPPQMYTSAQLN-------------GFENP
NcSho1  111 NNLIYS-GVASMEATAAGYILLSMVTIIWIFYFGSAPSAVPRAYIDSFALTKESTLPAHHMSRQTMNHNGLSSPNAYGSYNMRPETSASGLQPPQMYTGQLN-------------GLENP
AnSho1   92 NSMFNSKSTSSFQAAGAGFILLCMVNIVWTFYFGSAPQAKHRGFIDSFALNKENQGSYGANRPMSSAYG------------ARPETTTSR--PQMYTSAQLN-------------GFETS
ScSho1  112 TNLVYADGPK-KAAASAGVILLSIINLIWILYYGGDNASPTNRWIDSFSIKGIRPSPLENSLHRARRRGNRNTTPYQN-NVYNDAIRDSGYATQFDGYPQQQPSHTNYVSSTALAGFENT
CaSho1   90 NNLIYNSNSAGNLCCAAGCILLSILNLIWILYFGGHPESPTNQFIDSFSLRGQGHEQLGSGSHNHNANNANNNIPIGAGNAIIGKGEMSPYNDRFAASGVNQPTSESLRLASGPQMGNGP

MoSho1  199 SPVNGMRNSGAPPSGFPTTPGPASGLPKTTTPPAGGAA----------------------------------------------------------------------DAEIVPPTEYPY
NcSho1  217 ARQS------QIPQGFSSNNIPKP------------QG----------------------------------------------------------------------EGEIVPPTEYPY
AnSho1  185 SPVSG---YHGGAPGETRSPSQARFTSLGGPNASNPDT----------------------------------------------------------------------VGEIPPPTEYPY
ScSho1  230 QPNTSEAVNLHLNT-LQQRINSASNAKETNDNSNNQTNTNIGNTFDTDFS--------------------------------------------NGNTETTMGDTLGLYSDIGD-DNFIY
CaSho1  210 FTTTGAIINPNLQQPLSDSIGGSAHHTPINVNNNNNNNNNTGYMTSSHLTGLENFSSPHVPGSGTGAGLGVGAGRDLTHNSNGGGGSGGGPASANNSNNTNKRNTIYTDSETGTGITFRY

SH3
MoSho1  249 RAKAIYTYEANPDDANEISFSKHEILEVSDVSGRWWQARKETGETGIAPSNYLILL--------
NcSho1  249 RAKAIFSYEANPDDANEISFSKHEVLEISDVSGRWWQARKENGETGIAPSNYLILL--------
AnSho1  232 KAKAIYKYEANPEDANEIGFEKGEELEVSDVSGRWWQARKANGETGIAPSNYLILL--------
ScSho1  304 KAKALYPYDADDDDAYEISFEQNEILQVSDIEGRWWKARRANGETGIIPSNYVQLIDGPEEMHR
CaSho1  330 KAKALYSYDANPDDINEISFVKDEILEVDDIDGKWWQARRANGQVGICPSNYVKLLDT------
