# Supplementary material for: Multiple Plant Surface Signals are Sensed by Different Mechanisms in the Rice Blast Fungus for Appressorium Formation
Source: PLoS Pathog. 2011 Jan 20;7(1):e1001261. doi: 10.1371/journal.ppat.1001261 (PMC3024261; doi:10.1371/journal.ppat.1001261)
Supplement: Figure S2 — Nucleotide sequence of the MoMSB2 promoter region. Two PRE-like sequences were underlined. Two putative TCS elements were shaded in gray. (0.02 MB DOC) [file ppat.1001261.s002.doc]

GCTGGTTTTTTGGCCGGTTGGATTTCTTCTCTCGAACGCGAACCTGGAAGGACCCTTGGGAAAGTCATCGCGAGCTCACGGGAGCCTCCATGCCAAGCTCAGTTCCCTCATGAGACCCCAGACGCGACGACGGATCTGAAGGACCATTCCGAACCCTCTCGCAGACGAATTTTCGTCGATACGCTCTTTTAAAAAACCACCTAGATACACGATTAAGCCCTGGTGCTCATACGTCCAGTGAGTTGCCCATCAGCCGACGACGAGGGAGGACGATATATAGGAGTGTGAGGCGGAGGAAGCCGCATCTGTCAGGCGGGGCTTGAATTGTGCTTTCACCGCTGCATGAGCAGGCTCAGTAAGGGGCAGGCAAAGATATGGGCGGCTTCTGGGTTACCGAGTGCCAACATCCGCATCTTGAAGTCGATCCGCGGAGATTTTGCCATGCAGCGGACGATGCATTGCACACTACAGAGGGGGGTTCCATGGGCAATGGCTAACAACCGGTGGTCTTTGTCCAGCTCACTTACACTCGTTCAGTGACATCTTGTCAACCACATTCGTTTTCAATAAATCACCACAATGTGAGTCTGGCTGGTCTTGCTGCAGTTGAACTTGGTTGACAAAGGTTCTTCACCACCAGAGCAAACTTGCCAATCGTCTTTGCCACCCACGACCGAACAACTCGCATGTCATTCTAGGCTATTGGTCATCAGAACAACCGAAAACAGC
