# Supplementary figures and images for: Multiple Plant Surface Signals are Sensed by Different Mechanisms in the Rice Blast Fungus for Appressorium Formation
Source: PLoS Pathog. 2011 Jan 20;7(1):e1001261. doi: 10.1371/journal.ppat.1001261 (PMC3024261; doi:10.1371/journal.ppat.1001261)

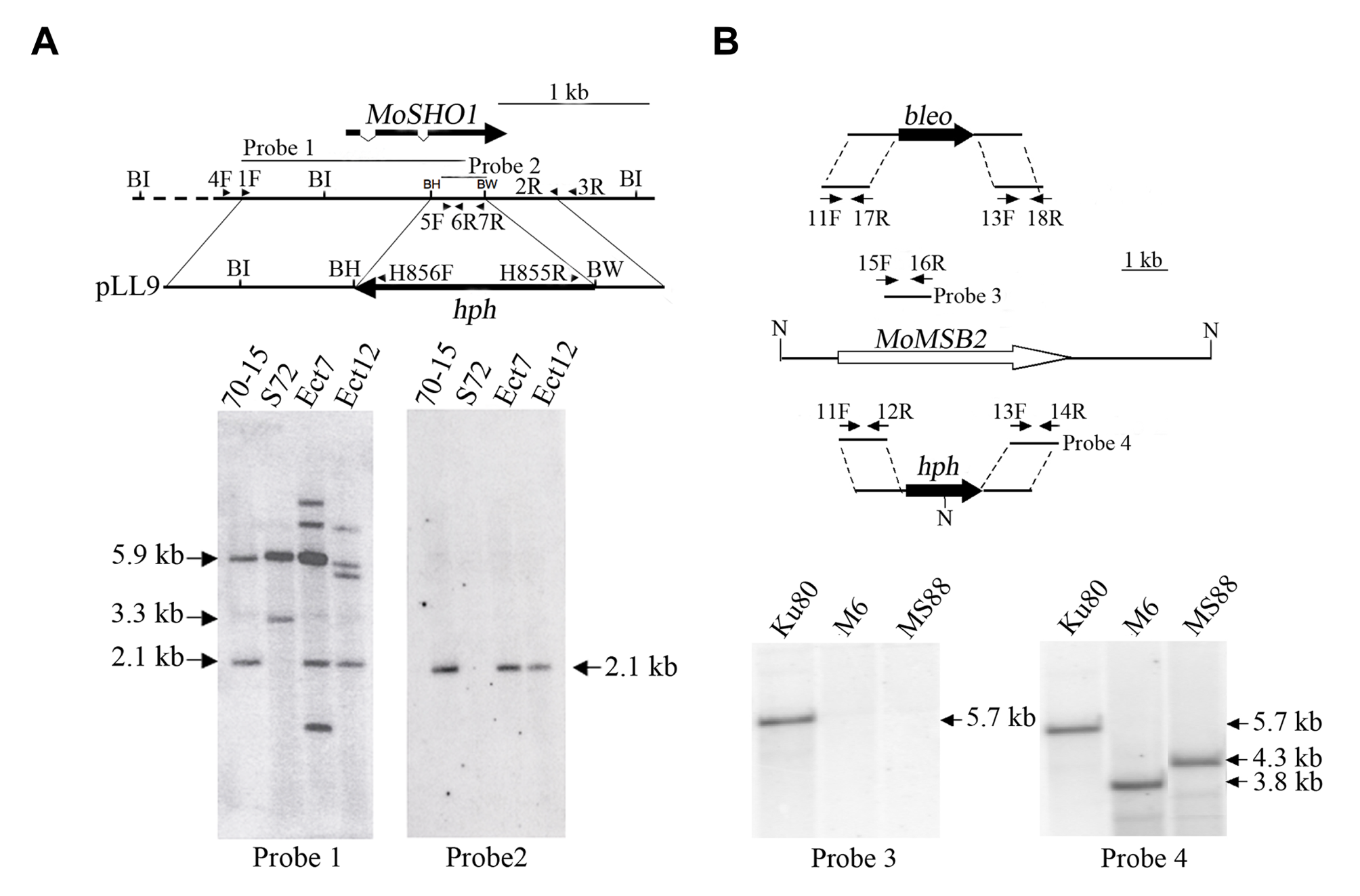

Supplement: Figure S3 — The Momsb2 and Mosho1 deletion mutants. A. The MoSHO1 gene replacement event and Southern blot analysis. Genomic DNA samples of 70-15 (WT), S72 (Mosho1), Ect7, and Ect12 (ectopic) were digested with BclI. The blot on the left was hybridized with probe 1 amplified with 1F and 6R. On the right was the same blot stripped and re-hybridized with probe 2 amplified with 5F and 7R. B. The MoMSB2 gene replacement events and Southern blot analysis of the Momsb2 (M6) and Mosho1 Momsb2 (MS88) mutants. When hybridized with a fragment of the MoMSB2 gene (probe 3), the wild-type 5.7-kb NcoI band was absent in mutants M6 and MS88. When hybridized with a downstream fragment of MoMSB2 (probe 4), mutants M6 and MS88 lacked the wild-type 5.7-kb band but had the expected 3.8-kb and 4.3-kb bands, respectively. (0.70 MB TIF) [file ppat.1001261.s003.tif]

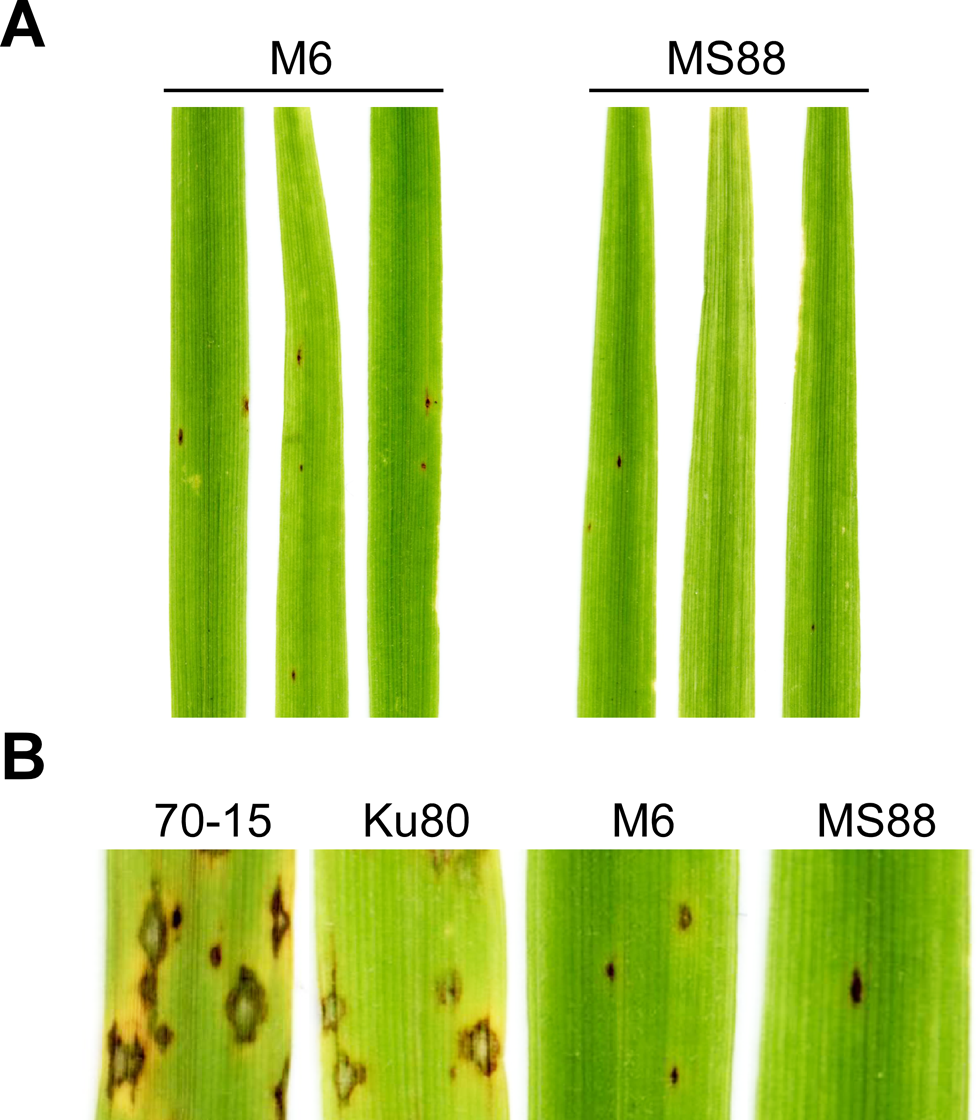

Supplement: Figure S4 — Lesions formed by the Momsb2 and Mosho1 Momsb2 mutants on rice leaves. A representative leaf tips sprayed with 5×10^4^ conidia/ml conidia from the Momsb2 and Mosho1 Momsb2 mutants. Typical leaves were photographed 7 dpi. B. Close view of lesions caused by the Momsb2 and Mosho1 Momsb2 mutants. (1.18 MB TIF) [file ppat.1001261.s004.tif]

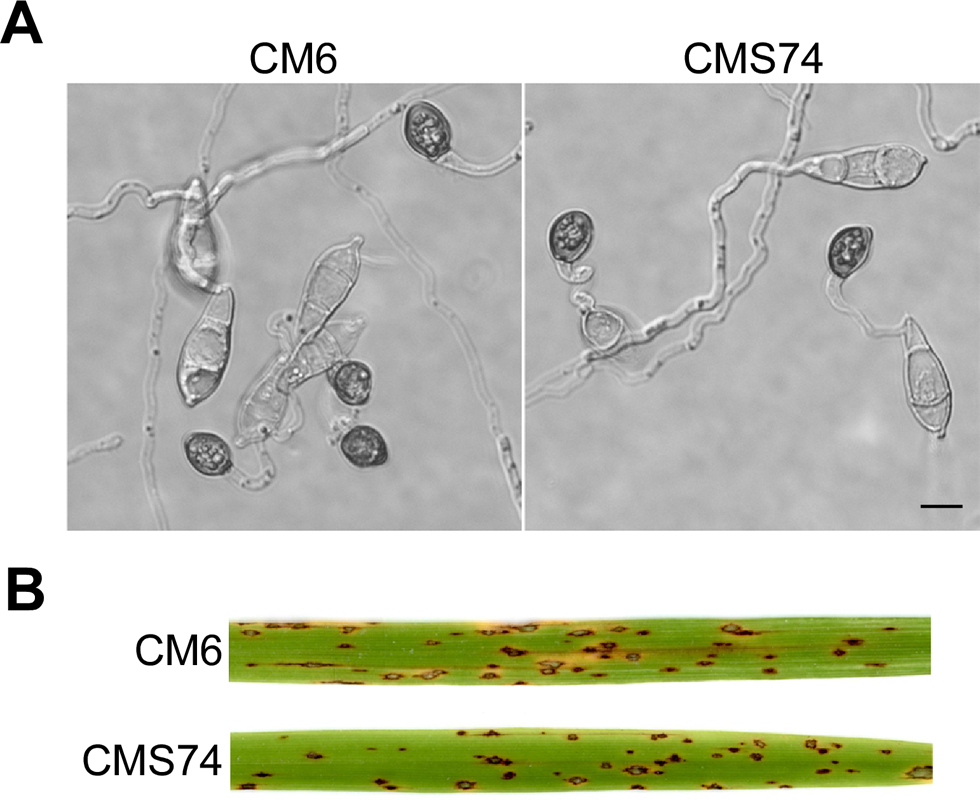

Supplement: Figure S5 — Appressorium formation and plant infection assays with the complemented transformants. A. Melanized appressoria formed by transformants CM6 (Momsb2/MoMSB2) and CMS74 (Mosho1 Momsb2/MoSHO1 MoMSB2) on hydrophobic surfaces. Bar = 10 µm. B. Blast lesions formed on rice leaves inoculated with CM6 and CMS74. (0.57 MB TIF) [file ppat.1001261.s005.tif]

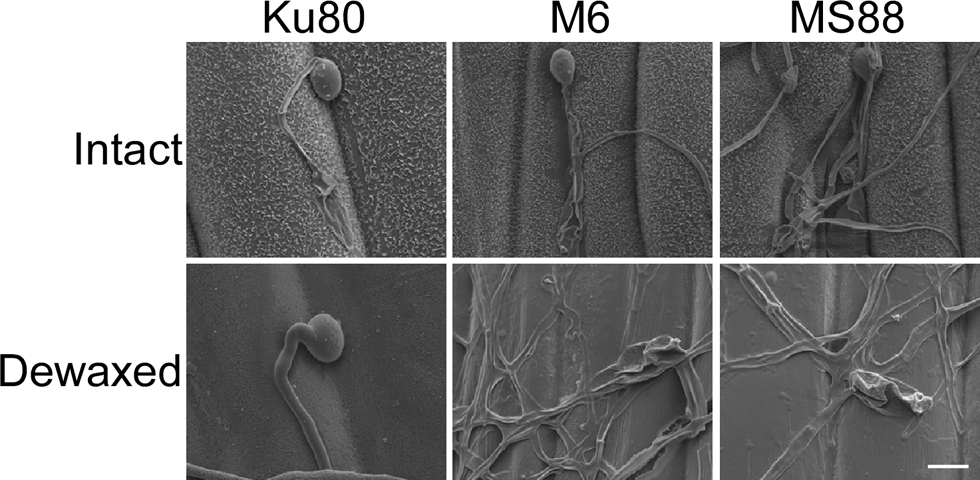

Supplement: Figure S6 — Barley leaves were inoculated with strains Ku80, M6 (Momsb2), and MS88 (Momsb2 Mosho1) and examined under SEM. The mutants formed appressoria on intact barley leaves (upper panels) but not on de-waxed leaves. Bar = 10 µm. (0.50 MB TIF) [file ppat.1001261.s006.tif]

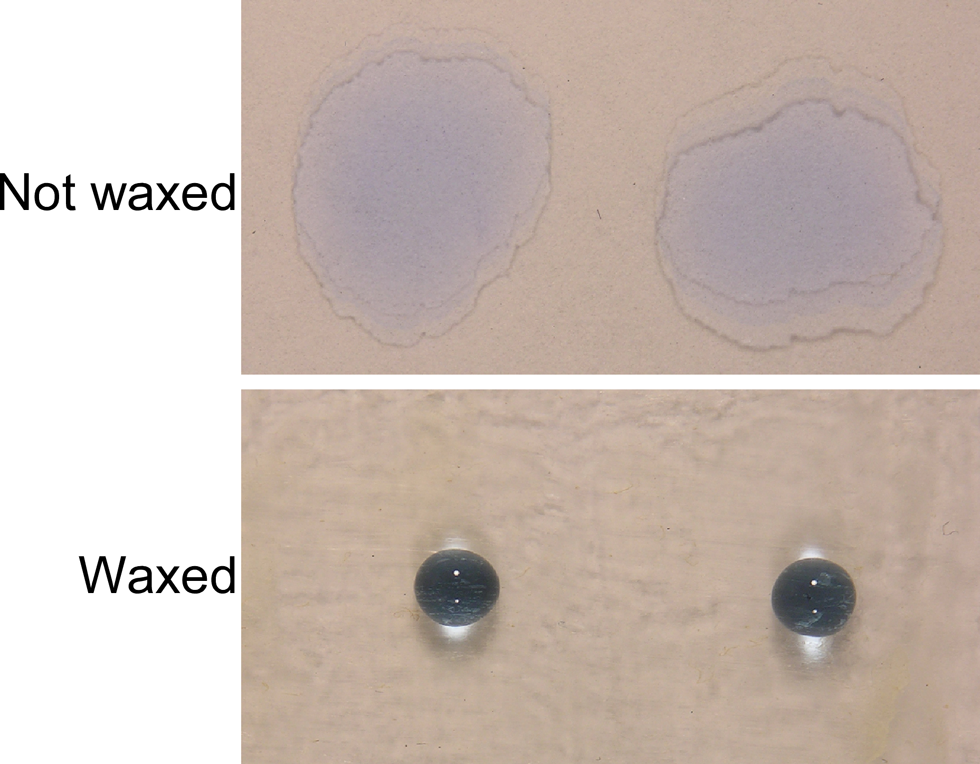

Supplement: Figure S7 — Surface hydrophobicity of waxed microscope glass slides. Drops of 20 µl of 0.02% bromophenol blue (BPB) in distilled water were placed onto the surface of glass slides that were untreated or coated with bee waxes. (0.85 MB TIF) [file ppat.1001261.s007.tif]

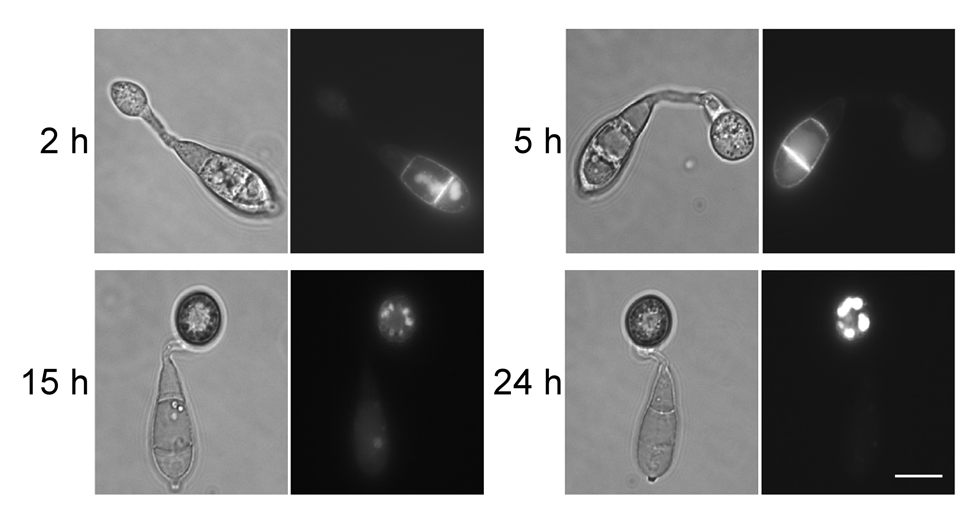

Supplement: Figure S8 — Time course assays for the expression and localization of MoMsb2-eGFP during appressorium formation. Representative images were presented for each time point (labeled on top). Bar = 10 µm. The same fields were examined under DIC (left) and epifluoresence microscopy (right). (0.28 MB TIF) [file ppat.1001261.s008.tif]
