# Supplementary material for: Multiple Plant Surface Signals are Sensed by Different Mechanisms in the Rice Blast Fungus for Appressorium Formation
Source: PLoS Pathog. 2011 Jan 20;7(1):e1001261. doi: 10.1371/journal.ppat.1001261 (PMC3024261; doi:10.1371/journal.ppat.1001261)
Supplement: Table S1 — PCR primers used in this study. (0.05 MB DOC) [file ppat.1001261.s009.doc]

**Table S1. PCR primers used in this study.**

| **Primer** | **Sequence (5΄→3΄)** | **application** |
| --- | --- | --- |
| 1F | tgaagccaaccctaggaa | *Mosho1* deletion |
| 2R | aggaaaccacttgcagca | *Mosho1* deletion |
| 3R | actagaagctgaaccaaa | *Mosho1* deletion |
| 4F | cctgtcgaaatcccaaag | *Mosho1* deletion |
| 5F | gcaaggagtcaacgagca | *Mosho1* deletion |
| 6R | ttgcgcatgccattgaca | *Mosho1* deletion |
| 7R | gtacgtgtagatggcctt | *Mosho1* deletion |
| H856F | gtcgatgcgacgcaatcgt | *Mosho1* deletion |
| H855R | gctgatctgaccagttgc | *Mosho1* deletion |
| HPH5F | ggcgtacgctggagctagtggaggtca | *Mosho1* deletion |
| HPH4R | cgggatcccggtcggcatctactctatt | *Mosho1* deletion |
| 11F | cgactgttcggcgacaaagactcttcc | *Momsb2* deletion |
| 12R | atatggccggccggcaagtttgctctggtggtgaagaac | *Momsb2* deletion |
| 13F | aattggcgcgccgctactcgccatacccacatcca | *Momsb2* deletion |
| 14R | cagctcagctaccaagggataacgttg | *Momsb2* deletion |
| Sho1F | atgtaagcttagagacacagtcaagttccgctggt | Complementation of yeast *sho1* mutant |
| Sho1R | attaggtaccgccatgaaaagcgctatatgttcat | Complementation of yeast *sho1* mutant |
| MGFPF | cactatagggcgaattgggtactcaaattggttcgccggtacgccactattatcctgggtt | MoMsb2-GFP fusion |
| MGFPR | caccccggtgaacagctcctcgcccttgctcacgttccagccgagtgagttctcctgtgc | MoMsb2-GFP fusion |
| ∆SSR1 | cgcgacaaatgccacggccaatttcgag | *MoMSB2*^∆SS^ |
| ∆SSF2 | ctcgaaattggccgtggcatttgtcgcgatgcctcggaaagtatatgatgggcctctc | *MoMSB2*^∆SS^ |
| ∆S1TR1 | ttcgctagcaggagtcgtcgtgttggc | *MoMSB2*^∆5STR^ |
| ∆S1TF2 | gccaacacgacgactcctgctagcgaaggtattctcatcgctcccaccggtgtc | *MoMSB2*^∆5STR^ |
| ∆S2TR1 | cacgacaccggtgggagcgatgagaat | *MoMSB2*^∆3STR^ |
| ∆S2TF2 | attctcatcgctcccaccggtgtcgtgaactccaatgacgactggctcccgacc | *MoMSB2*^∆3STR^ |
| ∆STRF2 | gccaacacgacgactcctgctagcgaaaactccaatgacgactggctcccgacc | *MoMSB2*^∆STR^ |
| ∆HMHR1 | ccagtcgtcattggagttggcgctgct | *MoMSB2*^∆HMH^ |
| ∆HMHF2 | agcagcgccaactccaatgacgactggttggtgaacccggctatcgacatcct | *MoMSB2*^∆HMH^ |
| ∆CDR | caccccggtgaacagctcctcgcccttgctcacgccagcagttcgagcagagttgcccg | *MoMSB2*^∆CT^ |
| Msb2QF | catcctccctggagctactctt | MoMSB2 qRT |
| Msb2QR | ttggtgctgttgttgtcgttg | MoMSB2 qRT |
| AQF | ccatgtaccctggtctttcg | MoActin qRT |
| AQR | ttcgagatccacatctgctg | MoActin qRT |
