# Supplementary material for: Multiple Plant Surface Signals are Sensed by Different Mechanisms in the Rice Blast Fungus for Appressorium Formation
Source: PLoS Pathog. 2011 Jan 20;7(1):e1001261. doi: 10.1371/journal.ppat.1001261 (PMC3024261; doi:10.1371/journal.ppat.1001261)
Supplement: Table S2 — Appressorium formation on intact and de-waxed rice leaves. (0.03 MB DOC) [file ppat.1001261.s010.doc]

**Table S2. Appressorium formation on intact and de-waxed rice leaves.**

| **Strain** | **Intact rice leaves (%)^a^** | **De-waxed rice leaves (%)** |
| --- | --- | --- |
| Ku80 | 99.3±1.1 | 83.5±5.0 |
| M6 | 68.7±7.3 | 6.5±2.2 |
| S72 | 90.1±3.6 | 63.9±5.8 |
| MS88 | 57.7±8.4 | 3.2±1.6 |

**^a^** Percentage of germ tubes formed appressoria.
